# Study of the Patterns of DNA Methylation in Human Cells Through the Prism of Intra-Strand DNA Symmetry

**DOI:** 10.3390/ijms26199504

**Published:** 2025-09-28

**Authors:** Zamart Ramazanova, Aizhan Alikul, Dinara Begimbetova, Sabira Taipakova, Bakhyt T. Matkarimov, Murat Saparbaev

**Affiliations:** 1 National Laboratory Astana, Nazarbayev University, Astana 010000, Kazakhstan; zamart.ramazanova@nu.edu.kz (Z.R.); dinara.begimbetova@nu.edu.kz (D.B.); 2 Department of Molecular Biology and Genetics, Faculty of Biology and Biotechnology, Al-Farabi Kazakh National University, Almaty 050040, Kazakhstan; aliqul.ayzhan@gmail.com (A.A.); sabira.taipakova@gmail.com (S.T.); 3 Scientific Research Institute of Biology and Biotechnology Problems, Al-Farabi Kazakh National University, Almaty 050040, Kazakhstan; 4 Faculty of Information Technology, L.N. Gumilev Eurasian National University, Astana 010000, Kazakhstan; 5 Groupe «Mechanisms of DNA Repair and Carcinogenesis», CNRS UMR9019, Université Paris-Saclay, Gustave Roussy Cancer Campus, F-94805 Villejuif Cedex, France

**Keywords:** DNA methylation, 5-methylcytosine, epigenetics, intra-strand DNA symmetry, Chargaff’s Second Parity Rule, cell differentiation, human cell types

## Abstract

Cellular organisms store heritable information in two forms, genetic and epigenetic, the latter being largely dependent on cytosine methylation (5mC). Chargaff’s Second Parity Rule (CSPR) describes the nucleotide composition of cellular genomes in terms of intra-strand DNA symmetry. However, it remains unknown whether DNA methylation patterns display intra-strand DNA symmetry. Computational analysis was conducted of the DNA methylation patterns observed in human cell lines and in tissue samples from healthy donors. Analysis of 5mC marks in mutually reverse-complementary pairs of short oligomers, containing CpG dinucleotide in the middle, revealed deviations from CSPR and methylation asymmetry that can be observed for two non-overlapping mirror groups defined by CpG methylation values. Deviations from CSPR, together with combinatorial probabilities of pattern distributions and computer simulations, highlight the non-random nature of methylation processes and enabled us to identify specific cell types as outliers. Further analysis revealed a compensatory methylation asymmetry that reduces deviations from intra-strand symmetry and implies the existence of strand-specific methylation during cell differentiation. Among six pairs of reverse-complementary tetranucleotides, four pairs with specific sequence motifs display pronounced methylation asymmetry. This mirror asymmetry may be associated with chromosome folding and the formation of a complex three-dimensional landscape.

## 1. Introduction

Epigenetic information refers to the ability of cells to maintain specific gene expression states and chromatin configurations across cell divisions—and in some cases across generations—without alterations in the underlying DNA sequence [1,2]. The transfer and storage of this information are largely mediated by epigenetic marks on DNA and histones, which, in response to morphogens and environmental cues, regulate gene activity by modifying chromatin structure.

One of the central mechanisms of epigenetic memory is DNA methylation, the post-replicative methylation of cytosine at the C5 position, producing 5-methylcytosine (5mC) in genomic DNA [3]. The stable propagation of DNA methylation patterns is ensured by DNA methyltransferase 1 (Dnmt1), which preferentially methylates hemimethylated CpG sites. This activity copies the parental methylation pattern onto the newly synthesized strand during DNA replication [4]. Two additional DNA methyltransferases, Dnmt3a and Dnmt3b, establish new methylation marks during development through de novo methylation at previously unmethylated sites [5].

During early embryogenesis, DNA methylation marks are globally erased, allowing the establishment of a totipotent epigenome [6]. After fertilization, active DNA demethylation mediated by TET enzymes helps reset the epigenetic landscape as embryonic cells transition from a totipotent to a pluripotent state and eventually into differentiated lineages [7,8]. In cancer, dysregulation of DNA methylation is a hallmark. Global DNA hypomethylation (arising from both active and passive demethylation) can activate transposable elements and promote genomic instability, while specific regions of the genome may undergo local hypermethylation, resulting in the silencing of tumor suppressor genes [9].

Genetic information is encoded in the linear DNA sequence through combinations of four chemical bases: adenine (A), thymine (T), cytosine (C), and guanine (G). The total number of possible combinations of a DNA sequence of length *N* is given by 4^N^, illustrating that DNA provides an immense reservoir of genetic information that can be efficiently stored, transferred, and decoded.

Biochemical and bioinformatic studies of natural DNA sequences have revealed intriguing geometric properties, most notably Chargaff’s Second Parity Rule (CSPR), also known as intra-strand DNA symmetry [10,11,12,13,14,15,16]. CSPR is a statistical property of cellular genomes, reflecting the near-equalities G ≈ C and A ≈ T within a single DNA strand [17]. Importantly, this symmetry extends beyond mononucleotides: for sufficiently long DNA sequences (greater than ~100 kb), CSPR holds for reverse-complementary pairs of short oligonucleotides (2–7-mers). For example, within one strand of cellular DNA, the trinucleotides **AGC** and **GCT** are present in nearly equal frequencies, |AGC| ≈ |GCT| [10,18,19]. CSPR is a universal property of cellular genomes, but there are exceptions. It does not apply to organelle genomes (mitochondrial and plastid DNA) smaller than ~20–30 kb, single-stranded viral DNA genomes, or any type of RNA genome [17].

Previously, we introduced the concept of DNA strand equivalence, which posits that each complementary single DNA strand within a chromosome is replicated approximately the same number of times over a sufficiently large number of cell divisions [20]. This strand equivalence provides a potential explanation for the maintenance of intra-strand DNA symmetry in cellular genomes: if both strands are inherited and replicated equally, symmetry is naturally preserved. Under conditions of no strand bias, random genetic variations in a population are expected to exhibit a statistical tendency toward balance. Specifically, each mutation should be “compensated” by an inverse complementary mutation elsewhere in the genome [13,14]. By analyzing SNP polymorphisms in human and mouse populations, we demonstrated that this compensatory mechanism can be confirmed by the fact that reverse-complementary mutation pairs (e.g., C → T and G → A) occur at similar frequencies on a single DNA strand. This phenomenon contributes to the maintenance of intra-strand DNA symmetry across entire segregating replicons [21].

During cell division, the faithful transmission of epigenetic information is crucial. Chromatin modifications are often copied using the parental single-stranded DNA as a template, in a manner analogous to DNA replication. Notably, the methylation of cytosine in CpG dinucleotides provides a relatively straightforward mechanism for propagating epigenetic marks. Because CpG sites are palindromic, the methylation pattern on the parent strand can be efficiently copied to the daughter strand during DNA synthesis. Here, we propose that the principle of DNA strand equivalence might also be extended to stable epigenetic modifications such as DNA methylation. Therefore, we hypothesize that DNA methylation patterns should conform to CSPR. Specifically, we expect that reverse-complementary oligonucleotide pairs will contain nearly identical numbers of 5mC marks—or conversely, nearly identical numbers of unmethylated cytosines—within CpG contexts.

One of the major challenges in modern biology is to understand how genetic and epigenetic programs coordinate embryonic development and morphogenesis. While genetic information can be interpreted as a linear text composed of four letters, decoding epigenetic information is a considerably more complex task. DNA methylation and histone modifications form highly dynamic, multilayered networks that are strongly context-dependent. For instance, it remains unclear how de novo DNA methyltransferases and histone-modifying enzymes accurately identify their target sites in the genome, particularly given the frequent absence of distinct DNA sequence motifs that would serve as binding sites—unlike the case for many transcription factors.

In the present study, we investigate whether intra-strand DNA symmetry is reflected in DNA methylation patterns in differentiated human cells. To address this, we analyzed two datasets:

(i) A dataset from the ENCODE Project (Encyclopedia of DNA Elements), specifically the RRBS (Reduced Representation Bisulfite Sequencing) data used to profile DNA methylation across various human cells and tissues (https://www.encodeproject.org/data (accessed on 14 May 2025)).
(ii) A recently published human methylome atlas, based on deep whole-genome bisulfite sequencing, which identifies cell-type-specific 5mC signatures across 39 cell types derived from 205 tissue samples of healthy donors [22].

Our analysis reveals that, in general, the numbers of methylated and non-methylated reverse-complementary oligonucleotide pairs are nearly equal (e.g., |AC^me^GA| ≈ |TC^me^GT|), but exhibit significant deviations from CSPR, when compared to human genome reference sequence. We further discuss the challenges of decoding epigenetic information and understanding how epigenetic writers, readers, and erasers interpret the primary DNA sequence to establish and maintain epigenetic marks in cellular genomes.

## 2. Results

### 2.1. Nucleotide Composition of Human Nuclear Genome Exhibits Intra-Strand Symmetry

One of the key features of the genomes of all cellular organisms, including humans, is their adherence to Chargaff’s second parity rule (CSPR), with deviations typically less than 1% from the mono- and oligonucleotide parity rule [17,19]. In this study, we examine the frequencies of tetra-nucleotides containing CpG in the middle (NCGN) across the human genome (GRCh37) and other datasets. Specifically, we analyzed the relative occurrences of six pairs of mutually reverse-complementary tetra-nucleotides (d(ACGA)/d(TCGT), d(ACGC)/d(GCGT), d(ACGG)/d(CCGT), d(CCGA)/d(TCGG), d(CCGC)/d(GCGG), d(GCGG)/d(CCGC)) and four self-complementary tetra-nucleotides within single-stranded DNA (ssDNA). As shown in Table 1A, the number of occurrences of d(ACGA) (denoted as |ACGA|) in one DNA strand of human chromosomes 1–22 is 1,499,090, which is nearly identical to the number of d(TCGT) (denoted as |TCGT|), which is 15,073,388, yielding a difference of only 0.55%. The remaining five pairs of reverse-complementary tetra-nucleotides are also present in nearly equal numbers, with differences of less than 1%, indicating that the human genome adheres to intra-strand DNA symmetry. Notably, the nucleotide composition of the human sex chromosomes also exhibits intra-strand DNA symmetry. In contrast, the human mitochondrial genome does not conform to CSPR and shows a strong nucleotide compositional bias (Table 1A).

We further analyzed the relative occurrence of reverse-complementary tetra-nucleotide pairs in two DNA methylome datasets: the ENCODE Project [23] and a recent publication describing a human methylome atlas (referred to as ATLAS) [22]. As shown in Table 1B, analysis of the ATLAS dataset, which covers the majority of CpG dinucleotides in the human genome, reveals nearly equal numbers of reverse-complementary tetra-nucleotides, where |NCGN| ≈ |N’CGN’|, with || denoting the total number of instances, N representing any nucleotide, and N’ representing the corresponding reverse-complementary counterpart. In contrast, analysis of the ENCODE dataset, which covers only a small portion of the human genome (~5%), reveals a significant deviation from CSPR. Five out of the six reverse-complementary tetra-nucleotide pairs exhibit more than a 1% difference, as shown in Table 1C.

Based on these analyses, we conclude that the human methylome ATLAS dataset closely reflects the major statistical features of the whole reference genome and can therefore be reliably used for further analysis (Table 1B). In contrast, the ENCODE dataset shows significant deviations from CSPR and may have limited utility (Table 1C). Nevertheless, to ensure broader representation, we have included the ENCODE dataset in our study.

### 2.2. Distribution of CpG Methylation Values

**Figure 1:**
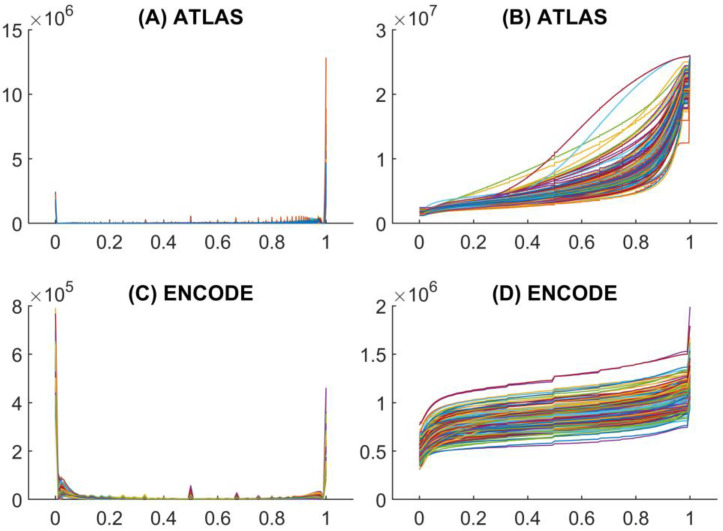
Distribution and cumulative distribution plots of methylation values (horizontal axis) against CpG dinucleotide counts (vertical axis) for all samples in the ATLAS (155 samples) and ENCODE (299 samples) datasets. (**A**) ATLAS distribution. (**B**) ATLAS cumulative distribution. (**C**) ENCODE distribution. (**D**) ENCODE cumulative distribution. Note that the different color of each lines in the panels corresponds to different cell samples.

As shown in Figure 1, the distribution of methylation values in both ENCODE and ATLAS datasets is highly heterogeneous, nonlinear, and unsmooth.

A major difference between the two datasets lies in their average sample sizes and in the relative counts of methylated versus non-methylated CpG sites. In the ENCODE dataset, the number of methylated sites is generally low, whereas in the ATLAS dataset it is typically very high compared to the number of non-methylated sites. This discrepancy reflects the distinct biological origins of the datasets: ENCODE predominantly includes cultured human cells grown in vitro or established cell lines, while ATLAS represents differentiated human cells derived from healthy donors. Notably, most ENCODE cell lines are human cancer cell lines, which are typically characterized by global hypomethylation (Figure 1C).

The heterogeneous nature of methylation value distributions complicates the grouping of samples according to their methylation levels. In the present study, we propose the following natural classification into three groups:

- ALL: includes all CpGs regardless of methylation value.
- VALZERO: includes only CpGs with a methylation value equal to 0 (100% non-methylated).
- VALONE: includes only CpGs with a methylation value equal to 1 (100% methylated).

Alternatively, one could classify CpGs using threshold values, for example: methylation < 0.2 (non-methylated), 0.2–0.8 (partially methylated), and >0.8 (fully methylated). However, such thresholds are arbitrary, and due to the nonlinear distribution of methylation values, the number of CpGs per group can vary greatly across samples.

For robust estimation of divergence from CSPR using the percentage difference metric, it is desirable to minimize variation in CpG counts within groups. This is difficult when sample sizes differ substantially between datasets. In fact, ENCODE samples have a mean CpG count of 1,989,310 with a standard deviation of 144,722 (7.3%), whereas ATLAS samples have a mean of 25,806,631 with a standard deviation of 133,669 (0.5%). Relatively balanced group sizes were achieved by splitting the datasets into halves or thirds based on methylation values (see Materials and Methods for details).

When dividing into halves, methylation values ranged from 0.554 to 1.0 for ATLAS (one is unacceptable value for halves) (mean = 0.89, SD = 0.062) and from 0 to 0.5 for ENCODE (zero is unacceptable value for halves) (mean = 0.052, SD = 0.08). Notably, 42 ENCODE samples and one ATLAS sample could not be evenly split into halves.

For division into thirds, the first cutoff ranged from 0.42 to 0.92 in ATLAS and from 0 (zero is unacceptable value for thirds) to 0.2 in ENCODE. The second cutoff ranged from 0.66 to 1.0 (one is unacceptable value for thirds) in ATLAS and from 0.07 to 0.96 in ENCODE. In total, only 266 out of 299 ENCODE samples and 2 out of 155 ATLAS samples could not be split into three valid groups.

### 2.3. Analysis of DNA Methylation Patterns in the Human Methylome Atlas Project

We analyzed data from the Human Methylome Atlas, which identifies unique 5mC residues across **39 cell types** derived from **205 tissue samples** of human donors [22]. Specifically, we compared the relative occurrence of methylated reverse complementary tetranucleotide pairs—d(AC^m^GA)/d(TC^m^GT), d(AC^m^GC)/d(GCGT), d(AC^m^GG)/d(CC^m^GT), d(CC^m^GA)/d(TC^m^GG), d(CC^m^GC)/d(GC^m^GG), d(GC^m^GG)/d(CC^m^GC)—where C^m^ denotes 5mC. In total, we examined **155** cell types (Table 2 and Supplementary Data Table S1).

For clarity, Table 2 presents results from 17 representative cell types. The first four rows from 2 to 5 summarize the statistics for all 155 cell types, showing maximum (MAX), minimum (MIN), average (AVG), and standard deviation (STD) values. Our analysis revealed a consistent bias from CSPR in 3–5 of the six tetranucleotide pairs, with deviations exceeding 1% between |NC^m^GN| and |N′C^m^GN′| counts (Table 2). Notably, the pair |AC^m^GA| and |TC^m^GT| showed the largest differences (3–6%) in most cell types. By contrast, the pairs |d(AC^m^GC)| vs. |d(GC^m^GT)| and |d(CC^m^GC)| vs. |d(GC^m^GG)| generally exhibited deviations below 1%. Importantly, in both the ATLAS and ENCODE datasets, tetranucleotide pairs flanked by two purines—either two adenines or an adenine and a guanine (|d(AC^m^GA)|, |d(AC^m^GG)| and |d(GC^m^GA)|)—showed the strongest deviations from parity (Table 2, Supplementary Data Tables S1 and S2).

In a preliminary assessment of the data from Table 2 and Supplementary Table S1, we conclude that DNA methylation patterns in the majority of human cell types studied show significant deviations (>1%) from intra-strand symmetry. This contrasts with the primary DNA sequence of the human genome, which conforms to CSPR—a key statistical feature of cellular genomes.

The relative occurrences of methylated reverse complementary tetra-nucleotide pairs for the ENCODE dataset are provided in Supplementary Data Table S2.

### 2.4. Analysis of Non-Methylated CpG Dinucleotides in the Human Methylome Atlas

As a complementary analysis, we examined the ATLAS dataset [22] to compare the relative occurrence of **non-methylated** reverse complementary tetranucleotide pairs— d(ACGA)/d(TCGT), d(ACGC)/d(GCGT), d(ACGG)/d(CCGT), d(CCGA)/d(TCGG), d(CCGC)/d(GCGG), d(GCGG)/d(CCGC)—where *C* in the CpG context represents unmethylated cytosine. Data from 155 cell types were analyzed (Table 3 and Supplementary Data Table S1). As in the previous analysis, the first four rows (rows 2–5 in Table 3) summarize the overall statistics across all 155 cell types, reporting maximum (MAX), minimum (MIN), average (AVG), and standard deviation (STD) values.

As shown in Table 3, non-methylated tetranucleotide pairs also exhibit significant deviations from CSPR, although generally to a lesser extent than methylated tetra-nucleotides. More than half of the cell types show near parity for at least three of the six pairs, specifically d(ACGC)/d(GCGT), d(ACGG)/d(CCGT), and d(CCGC)/d(GCGG). Consistent with our observations for methylated sequences, non-methylated tetra-nucleotide pairs flanked by two purines—either two adenines or an adenine and a guanine (|ACGA|, |ACGG|, and |GCGA|)—show the strongest deviations from parity.

To a first approximation, analysis of the data from Table 3 and Supplementary Data Table S1 suggests that patterns of non-methylated tetranucleotides in roughly half of the human cell types studied deviate significantly (>1%) from the intra-strand symmetry of the underlying nucleotide sequence. The relative occurrences of non-methylated reverse complementary tetra-nucleotide pairs for the ENCODE dataset are provided in Supplementary Data Table S2.

### 2.5. Analysis of the Distributions of CSPR Deviations Reveals Compensatory Role of Methylation Asymmetry in Mutually Reverse Complementary Patterns

As indicated by the DIFF% values, the divergence among samples is relatively large. An important question is whether this divergence reflects a normal distribution due to random variation, or whether it instead points to biological processes that cause systematic perturbations. To address this, we generated histograms with fitted normal distribution curves for all groups in both the ENCODE and ATLAS datasets (Supplementary Figures S1 and S2).

The distributions of DIFF% values do not resemble a normal distribution and, in fact, fail the one-sample Kolmogorov–Smirnov test at any reasonable significance level. This indicates that the observed variations are unlikely to arise from random fluctuations alone.

Histograms provide a visualization of data distributions across whole datasets or specific subsets of samples, depending on group definitions. The DIFF% metric measures the asymmetry between the counts of two reverse complementary patterns: the magnitude indicates the degree of deviation, while the sign indicates which pattern is more prevalent in the methylation data.

**Figure 2:**
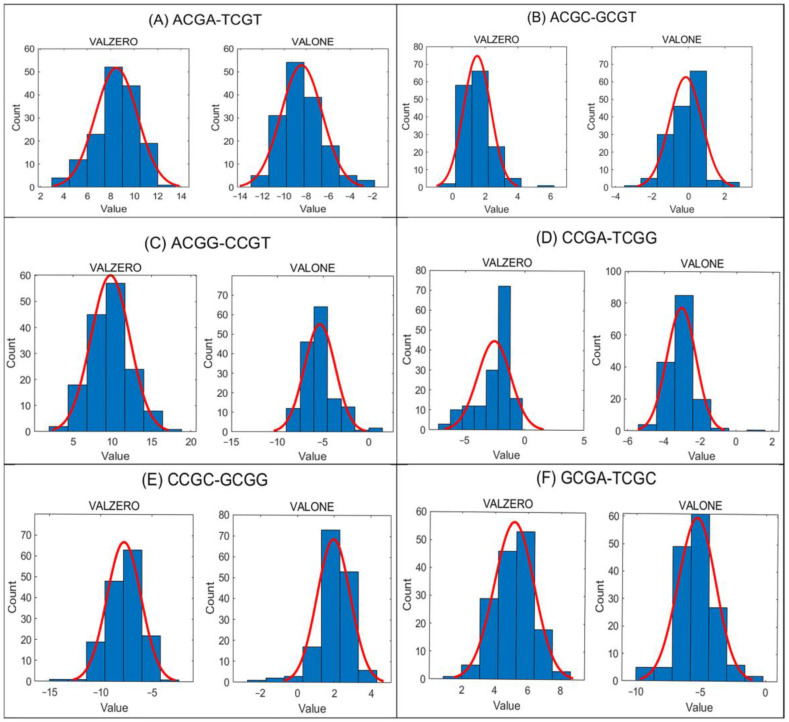
Histograms of DIFF% values (horizontal axis) versus number of samples (vertical axis) from the ATLAS dataset (155 samples), for the “VALZERO” and “VALONE” groups. DIFF% distributions are shown for the following tetra-nucleotide pairs: (**A**) ACGA–TCGT, (**B**) ACGC–GCGT, (**C**) ACGG–CCGT, (**D**) CCGA–TCGG, (**E**) CCGC–GCGG, (**F**) GCGA–TCGC.

In Figure 2, histograms with fitted normal distribution curves are shown for all tetranucleotide pairs in the VALZERO and VALONE groups from the ATLAS dataset.

The data reveal pronounced asymmetry for all tetra-nucleotide pairs flanked exclusively by purines or, in the reverse complementary counterpart, by pyrimidines: ACGA–TCGT, ACGG–CCGT, CCGC–GCGG, and GCGA–TCGC. Importantly, the sign of DIFF% differs consistently between the VALZERO and VALONE groups. In the ATLAS dataset, this pattern is observed in all 155 samples for ACGA–TCGT and GCGA–TCGC, in 153 samples for ACGG–CCGT, and in 149 samples for CCGC–GCGG. Such opposite signs between VALZERO and VALONE act in a compensatory manner when combined in the ALL group, thereby reducing the absolute DIFF% values.

For mixed purine–pyrimidine pairs (ACGC–GCGT and CCGA–TCGG), the observations differ. In the ATLAS dataset, DIFF% shows opposite signs between VALZERO and VALONE groups in only 77 out of 155 samples for ACGC–GCGT, and in just 1 out of 155 samples for CCGA–TCGG. Thus, ACGC–GCGT does not display consistent methylation asymmetry, while CCGA–TCGG largely maintains methylation symmetry across groups.

The above analysis of the distributions of DIFF% values in a sample reveals mirror asymmetry depending on methylation value of cytosines, for mutually reverse complementary pairs of tetra-nucleotides. For each sample, methylation asymmetry is observed across different groups (e.g., VALZERO and VALONE) (Figure 2). Notably, each tetra-nucleotide is located at distinct genomic positions and exhibits a range of experimentally measured cytosine methylation values. A given CpG with its methylation value in the sample belongs only to one group and cannot be simultaneously occur in two or more different groups.

This methylation asymmetry reveals a significant statistical feature in the parameters associated with cytosine methylation values, which is linked to CSPR. For example, in one group (e.g., VALONE), if a specific tetra-nucleotide pair containing a CpG dinucleotide—such as ACGA and its reverse complement TCGT—demonstrates higher number of methylated TCGT compared to that of ACGA, the opposite pattern is often observed in another group (e.g., VALZERO), where the reverse complement (ACGA) is more frequently unmethylated than TCGT. This compensatory pattern of methylation asymmetry is analogous to the compensatory role of CSPR in the strand bias observed in GC/AT skew [24].

In the group “ALL”, the maximum by absolute value DIFF% over all samples is lower as compared with the other groups, emphasizing compensatory nature of the methylation values distributions in other groups. If in a subset of ALL (e.g., VALONE) for a pair of methylated tetra-nucleotides (e.g., GCGA-TCGC) we observe a bigger value of DIFF% than in a whole set (ALL) for the pairs of mutually reverse complementary tetra-nucleotides, then in a different subset (e.g., VALZERO) we will observe DIFF% with a different sign for the same pair (Figure 2A,C,E,F).

More generally, if a subset of methylated CpG sites shows a larger DIFF% than the entire dataset for a given reverse complementary pair, the complementary subset (relative to the whole) will exhibit a DIFF% of opposite sign. This reveals an important feature of the methylation processes, where complementary subsets balance one another.

A similar compensatory phenomenon is well known in DNA sequence organization and described as nucleotide skew [25]. Locally, in a chromosomal region, the frequency of a pattern (X) may exceed that of its reverse complement (X′), yet at the genome-wide scale these differences are reduced. In prokaryotes, for example, nucleotide skew typically extends over half the genome around the origin of replication.

**Figure 3:**
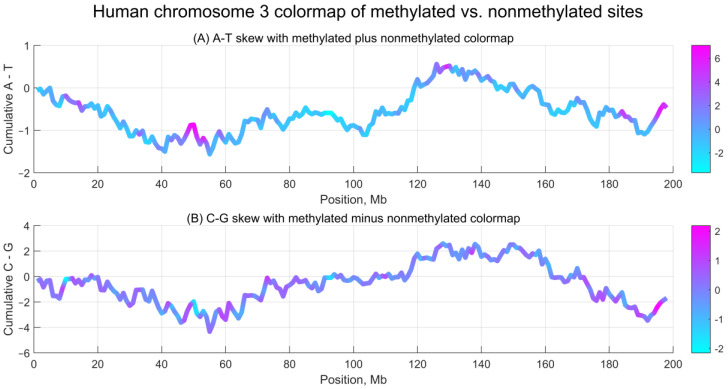
Chromosome 3 colormaps of normalized sums and differences for methylated and non-methylated sites compared with nucleotide A–T and C–G skews. Nucleotide skew is plotted on the vertical axis, and genomic position (in Mb; centromere at position 91) is on the horizontal axis. (**A**) A–T nucleotide skew with colormap of normalized methylated plus non-methylated counts. (**B**) C–G nucleotide skew with colormap of normalized methylated minus non-methylated counts.

To investigate whether methylation asymmetry exhibits similar properties, we divided chromosome 3 into 1 Mb intervals and computed the numbers of methylated (value = 1) and non-methylated (value = 0) CpG sites. Because these counts differ by orders of magnitude (see Figure 1), we normalized both data series to mean 0 and standard deviation 1, then calculated their sum and difference, mapping the results to nucleotide skew profiles. Figure 3 shows the normalized difference between methylated and non-methylated CpG sites along chromosome 3, aligned with A–T and C–G nucleotide skew.

As shown in Figure 3, the distributions along chromosome 3 reveal both hotspots and regions of stability: methylated plus non-methylated sites align with A–T skew, while methylated minus non-methylated sites align with C–G skew. However, no strong correlations were observed between nucleotide skew and methylation asymmetry. Pearson correlation coefficients between the normalized sums or differences and the two skews are below 18% in absolute value. By contrast, the correlation between methylated and non-methylated series is 81.4%, and between A–T and C–G skews is 87.3%.

Notably, similar to DNA nucleotide skew, methylation asymmetry shows a **continuous rather than dispersed (heterogeneous)** character across the genome, with smoothly varying accumulated values when computed over sufficiently large intervals.

### 2.6. Samples Outliers

The common (base-10) logarithms of combinatorial probabilities were computed for all samples and for all pairs of reverse complementary patterns (see Supplementary Data Tables S1 and S2). These computations were performed in double precision; therefore, the logarithmic values are approximate. To assess accuracy, we recalculated results for one representative sample, “GSM5652298_Blood-T-Naive-CD8-Z0000041H”, using arbitrary-precision arithmetic. The comparison confirmed that double-precision computations are exact up to six digits after the decimal point.

Using normal distribution fits for both DIFF% values and combinatorial probability distributions across all groups in the ATLAS and ENCODE datasets, we identified outlier samples as those differing from the mean by more than three standard deviations (3σ). Details of these outliers are provided in Supplementary Data Table S3.

For the ATLAS dataset, the DIFF% distribution revealed only 13 distinct outlier cell types. These cell types and their group assignments are listed in Table 4. The combined set of ATLAS outliers, considering both DIFF% and combinatorial probability distributions, includes 24 cell types in total (Supplementary Data Table S3).

For the ENCODE dataset, the combined set of outliers (DIFF% and/or combinatorial probability distributions) includes 92 samples, several of which are nearly identical.

Sample outliers are defined as those lying at the borders of the overall sample distribution according to numerical criteria, such as the DIFF% metric or combinatorial probabilities. Importantly, there is no need to fit statistical distributions to histograms; instead, all samples (listed in Supplementary Data Tables S1 and S2) can simply be sorted by numerical criteria, and a specified number of samples may be selected from either the top or bottom of the list. The resulting set of outliers includes well-characterized cell types with known DNA methylation patterns, such as Neurons, Cortical Neurons, Blood T Naive CD8 cells, Kidney Glomerular Podocytes, etc.

### 2.7. Random Sampling Simulation of Sample Outliers Demonstrate That the Process of DNA Methylation Is Not Random

An important question is whether the CpG distributions observed in outlier samples are truly exceptional with respect to DIFF% values for reverse complementary pairs, or whether similarly extreme DIFF% values could arise by chance through random sampling of CpGs with the same total counts as in the sample. To address this, we performed computer simulations for every outlier sample (see Materials and Methods for details). For the ALL group, simulations are not applicable because the group by definition includes all CpG sites from a sample, leaving no alternative combinations.

In the ENCODE dataset, for all outlier samples (except those in the ALL group), random sampling consistently produced sets of CpG sites with larger DIFF% values across all six mutually reverse complementary pairs.

In the ATLAS dataset, results differed by group. For VALONE and VALZERO, CpG distributions with larger DIFF% values were found in only two of the six outlier samples (both Bone marrow erythrocyte progenitors), and within fewer than 50,000 random iterations. For the HALF and THIRD groups, random distributions with larger DIFF% values were generally not identified within 50,000 iterations, except for the THIRD-MID sample “GSM5652274_Bone_marrow-Erythrocyte_progenitors-Z000000RF”, which was recovered after 32,206 iterations. In most other ATLAS outliers, simulations occasionally produced higher DIFF% values, but only for a subset of the six tetra-nucleotide pairs.

**Figure 4:**
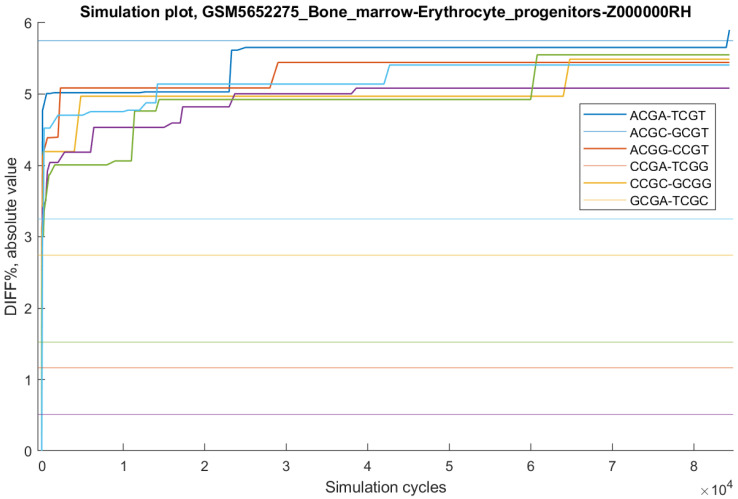
Simulation results for sample “GSM5652275_Bone_marrow-Erythrocyte_progenitors-Z000000RH”. Horizontal lines indicate the absolute DIFF% values observed in the sample for six mutually reverse complementary pairs—used here as simulation target values.

As an example, Figure 4 shows simulation results for the VALONE sample “GSM5652275_Bone_marrow-Erythrocyte_progenitors-Z000000RH”. For the reverse complementary pairs ACGC–GCGT, ACGG–CCGT, CCGA–TCGG, CCGC–GCGG, and GCGA–TCGC, the simulation identified random CpG sets with DIFF% values exceeding the target in fewer than 200 cycles. However, for the ACGA–TCGT pair (DIFF% = −5.75; combinatorial probability = 2.69 × 10^−4^), the simulation required 84,390 cycles. In independent runs, all targets were found in either 1856 or 46,512 cycles. Because random sampling is stochastic, the number of iterations required for success cannot be predicted in advance, but the expected value can be approximated as 1/probability—in this case, ~3717 cycles.

If the probability of an event is *p*, then the probability that the event occurs at least once within *N* iterations is 1 − (1 *− p*)*^N^*; for very small *p* this may be approximated as *p* × *N*. For example, with 500,000 random samplings, the expected probability of success is ~2 × 10^−6^, corresponding to a base-10 logarithm of about −5.7.

In the ENCODE dataset, for all outlier samples, the combinatorial probability associated with DIFF% values is greater than −5.74. Accordingly, random simulations succeeded in reaching the targets. By contrast, in the ATLAS dataset, most outlier samples contain reverse complementary pairs with DIFF% probabilities far below −6, and random sampling simulations did not succeed within 50,000 iterations.

### 2.8. Analysis of Inter-Relation Between DIFF% and Combinatorial Probabilities Reveal Nice Geometrical Patterns

It is important to note that DIFF% is calculated directly from the counts of two mutually reverse complementary patterns (*X* and *X*′) with their specific methylation states. In contrast, combinatorial probabilities account for the possible reallocation of methylation, i.e., the number of distinct ways in which *X* or *X*′ could be selected from the full set of observed patterns.

**Figure 5:**
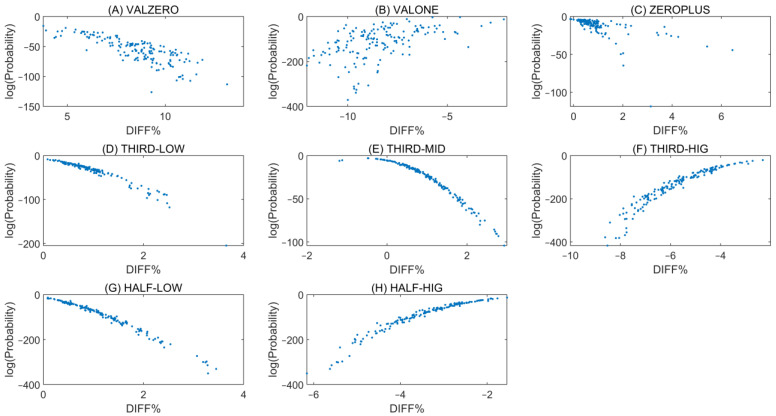
Scatter plot of DIFF% versus the base-10 logarithm of combinatorial probabilities for the ACGA–TCGT reverse complementary pair across groups: (**A**) VALZERO, (**B**) VALONE, (**C**) ZEROPLUS, (**D**) THIRD-LOW, (**E**) THIRD-MID, (**F**) THIRD-HIG, (**G**) HALF-LOW, and (**H**) HALF-HIG.

Figure 5 shows a scatter plot of DIFF% versus combinatorial probabilities for the ACGA–TCGT pair across all samples in all groups (excluding ALL).

There is no strict one-to-one relationship between DIFF% and combinatorial probabilities. The absolute Pearson correlation coefficients between these two measures for the ACGA–TCGT pair are as follows: VALZERO 80.1%, VALONE 50.0%, ZEROPLUS 60.4%, THIRD-LOW 96.5%, THIRD-MID 95.2%, THIRD-HIG 93.5%, HALF-LOW 98.7%, and HALF-HIG 95.0%.

Across all groups, samples with DIFF% values close to zero tend to exhibit higher combinatorial probabilities. This observation supports the expectation from Chargaff’s second parity rule, although important deviations remain. In particular, zero DIFF% does not always correspond to the most probable configuration. For example, the ACGG–CCGT pair, as well as additional pairs such as ACGC–GCGT and CCGC–GCGG, show systematic violations in both the ATLAS (Supplementary Figure S3) and ENCODE (Supplementary Figure S4) datasets.

## 3. Discussion

### 3.1. Divergences of Methylation Profiles from the CSPR

Intra-strand DNA symmetry, or CSPR, is a fundamental statistical property of the genomes of all cellular organisms. Remarkably, this symmetry has been preserved throughout billions of years of evolution, despite the vast diversity of species that have arisen from a single common ancestor. Genetics—the study of genes, genetic interactions, inheritance, the genetic code, and nucleotide composition—is strongly shaped by CSPR. We have recently proposed that intra-strand DNA symmetry underlies additive genetic interactions and contributed to the optimization of the standard genetic code [21]. Genetic information stored in the DNA sequence determines phenotype through transcription, translation, and genetic interactions, which rely on base pairing, the genetic code, and CSPR, respectively. In contrast, epigenetics examines heritable and reversible changes in gene expression that occur without altering the underlying nucleotide sequence of a gene or genome for example via DNA methylation. At present, it remains unclear how de novo DNA methylation and the erasure of 5mC marks are specifically targeted to certain genetic loci in the absence of a well-defined DNA sequence motif.

As we wrote above, in differentiated cells, the epigenetic chemical modifications are faithfully duplicated in each cell cycle, indicating that each parental single-strand DNA contains information in form of 5mC marks and histone modifications enabling the chromatin structures to propagate through semiconservative DNA replication. Therefore, each single-strand in a DNA duplex contains complete information to restore nucleotide sequence and epigenetic modifications implying strand equivalence. Based on these reflections, we suggest that nucleotide composition symmetry of genomic DNA might be echoed at the epigenetic level. To examine this, we analyzed the patterns of DNA methylation in human cells using two datasets: Human Methylome Atlas and Encode. Somewhat surprisingly, computational analysis of the two datasets revealed significant deviations of DNA methylation patterns in majority of human cells from CSPR (DIFF more than 1% of difference between methylated or non-methylated reverse complementary tetra-nucleotides), suggesting that either the transfer of epigenetic information during DNA replication and after cell division occurs in a fuzzy manner or the establishment of 5mC marks during cell differentiation occurs in asymmetric manner (Table 2 and Table 3, Tables S1 and S2). Furthermore, the distribution of methylation patterns for certain cell types deviates even more greatly from intra-strand DNA symmetry, suggesting possible involvement of strand asymmetric processes.

### 3.2. Methylation Asymmetry and CSPR

In the present study, we demonstrate the compensatory nature of methylation asymmetry when analyzing VALZERO (methylation value 0) and VALONE (methylation value 1) groups (Figure 2).

When analyzing the distributions of DIFF% values in the VALZERO and VALONE groups, it is important to note that CpG sites with methylation values strictly greater than 0 and less than 1 are not represented in the histograms (Figure 2). To account for the absence of these intermediate methylation values, we generated the THIRD and HALF group families (see Supplementary Figures S1 and S2), which partition the entire ALL group into nonoverlapping subsets that include all CpGs. The distributions of DIFF% values in these groups likewise reveal the compensatory nature of methylation asymmetry across the full range of methylation values of all CpGs.

Analysis of the distribution of the normalized differences between methylated and unmethylated CpG sites along chromosome 3 demonstrated continuous patterns of mutually reverse-complementary statistics over relatively long genomic intervals, extending to several megabases (Figure 3). Although the majority of CpG sites occur in an ungrouped, non-clustered manner across chromosomes, the normalized difference between methylated and unmethylated sites often changes sign at specific positions and shows continuous increases or decreases between these points. Across any sufficiently long interval of chromosome 3, CpGs with methylation values of 0 or 1, in fact, coexist with those exhibiting intermediate methylation levels. Interestingly, we sometimes observe a simultaneous increase or decrease in the number of CpGs with both fully methylated (value = 1) and fully unmethylated (value = 0) states. This seemingly paradoxical behavior can be explained by a hidden, continuous reduction or increase in the number of CpGs with intermediate methylation values.

Astronomically small probabilities for pairs of patterns can be misleading, as such events may still occur in reality. In our case, the combinatorial probability depends on four parameters (see Materials and Methods). At the genomic scale, the values of all four parameters are extremely large, reaching hundreds of millions. Consequently, the number of possible combinations for selecting large sets of patterns is astronomically high. We do not claim that pairs of patterns with combinatorial probabilities as low as 10^−400^ cannot occur, especially since similar patterns are observed in experimental data. Rather, we emphasize that it is practically impossible to encounter such rare combinations through random sampling (i.e., by brute force), because the expected number of iterations required far exceeds the total number of cell divisions within a human lifespan or any comparable biological process.

Methylation asymmetry represents a compensatory phenomenon in the distribution of methylation values between mutually reverse-complementary pairs, tending to minimize deviations from CSPR at the global chromosomal level. We propose that detailed characterization of different complementary subsets could uncover the hidden structure of methylation processes. 3.3. Possible Biological Origin of the Strand-Specific DNA Methylation

As long as the human genome—including every chromosome—adheres to CSPR when comparing the frequencies of short oligomers with length up to 7 nucleotides, including any random subset of CpG’s with flanking nucleotide sequences (NCGN) and their mutually reverse complementary counterparts then any such subset should reflect this symmetry. Many cellular processes, like replication and transcription, have strand asymmetric nature causing a local deviation from CSPR; thus DNA methylation may be associated with some cellular asymmetric processes. The question of why a particular pair of reverse-complementary patterns either exhibits methylation asymmetry to compensate for deviations from CSPR or instead conforms to CSPR remains to be investigated.

During embryogenesis, cells progressively lose pluripotency and acquire lineage-specific fates. This process of cell differentiation is guided by the establishment of stable epigenetic marks that lock in cell identity. DNA methylation together with histone modifications and chromatin remodeling are the main epigenetic marks that shape transcriptional programs. Notably, after fertilization, the paternal and maternal genomes undergo extensive reprogramming, through which most DNA methylation marks are erased, followed by a wave of de novo methylation during implantation. As cells differentiate, DNA methyltransferases (DNMT3A, DNMT3B) establish cell-type-specific methylation patterns, while DNMT1 maintains them through cell divisions. Based on these observations, we may propose that the asymmetric distribution of 5mC marks within single DNA strands take place during de novo methylation catalyzed by DNMT3A and DNMT3B. It is tempting to speculate that de novo application of 5mC marks occurs on already folded chromosomes that have a three-dimensional (3D) shape. Therefore, the methylation strand asymmetry appears because epigenetic writers act on a complex 3D surface.

In another hypothesis, we suggest that the blurred transfer of DNA methylation marks across cell divisions resembles the phenomenon of genomic imprinting in which only one copy of a gene is active, depending on whether they are inherited from the female or male parent [26]. In a similar way, we may hypothesize that during semiconservative replication, 5mC marks on each DNA strand are inherited in an asymmetrical manner, depending on whether it is a forward positive strand or reverse negative strand. This means that the 5mC mark in one of the parental DNA strands, for example, a forward strand, is passed to the daughter cell, whereas 5mC in the reverse strand is not, due to active or passive DNA strand-specific demethylation.

## 4. Materials and Methods

### 4.1. Datasets Used in the Study

1. ATLAS—155 samples from the DNA Methylation Atlas of Normal Human Cell Types, downloaded from: https://www.ncbi.nlm.nih.gov/geo/query/acc.cgi?acc=GSE186458 (accessed on 14 May 2025) [22].
2. ENCODE—299 samples from DNA Methylation by Reduced Representation Bisulfite Sequencing (RRBS) generated by ENCODE/HudsonAlpha and hosted by the UCSC Genome Browser. Data were downloaded from: https://hgdownload.cse.ucsc.edu/goldenPath/hg19/database/ (accessed on 14 May 2025) with related track information available at: https://genome.ucsc.edu/cgi-bin/hgTrackUi?db=hg19&g=wgEncodeHaibMethylRrbs (accessed on 14 May 2025) [27]. UCSC Genome Browser references are provided in [28,29,30].

Both datasets report the fraction of DNA molecules with cytosine methylation at CpG dinucleotides. The complete list of analyzed cell types with dataset-specific file identifiers is provided in the Supplementary Materials (Supplementary Data Table S4). Importantly, the ENCODE dataset represents human cell lines, whereas the ATLAS dataset comprises differentiated human cells isolated from healthy donors.

In this study, we used datasets from ENCODE and ATLAS, while cancer-related methylation datasets were not used. The ATLAS dataset was only partially analyzed (155 out of 253 samples) due to download limitations; however, we ensured that at least two samples were included for each cell type. The ENCODE dataset contains redundant samples that differ as binary files but are almost identical in their statistical properties.

Our main research findings are based on the analysis of the ATLAS dataset, with the ENCODE dataset serving as an illustrative counterpart. Cancer-related methylation datasets were not included in the analysis because of controlled access restrictions.

### 4.2. Data Processing

For each sample, we extracted the genomic position, methylation value, and flanking nucleotide sequences (i.e., CpG patterns with one flanking nucleotide on each side) for every CpG dinucleotide with an exact genomic coordinate (excluding sites reported within ranges). In total, we retrieved 362,795,553 CpG dinucleotides from the ENCODE dataset and 4,000,027,839 CpG dinucleotides from the ATLAS dataset.

There are 16 possible tetranucleotides containing a CpG dinucleotide at the center. Among these, 4 are self–reverse complementary (i.e., identical to their reverse complement: ACGT, CCGG, TCGA, GCGC), while the remaining 12 form six reverse complementary pairs: ACGA-TCGT, ACGC-GCGT, ACGG-CCGT, CCGA-TCGG, CCGC-GCGG, GCGG-CCGC.

For each sample, CpG methylation data were stratified into the following groups:

1. ALL—all CpG sites in the sample (Table 1; Supplementary Data Tables S1 and S2).
2. VALZERO—CpG sites with a methylation value of 0 (100% non-methylated) (Supplementary Data Tables S1 and S2).
3. VALONE—CpG sites with a methylation value of 1 (100% methylated) (Supplementary Data Tables S1 and S2).
4. HALF (HALF-LOW and HALF-HIGH)—CpG sites split into two approximately equal groups based on methylation value. For each sample, a threshold was determined that divided CpG sites into two sets: HALF-LOW (methylation ≤ threshold) and HALF-HIGH (methylation > threshold) (Table 2 and Table 3; Supplementary Data Tables S1 and S2). Note: 42 samples in the ENCODE dataset and 1 sample in the ATLAS dataset could not be divided into two groups by methylation value.
5. THIRD (THIRD-LOW, THIRD-MID, THIRD-HIGH)—CpG sites split into three approximately equal groups by methylation value. Two thresholds were determined for each sample: sites with methylation ≤ first threshold were assigned to THIRD-LOW, sites with values between the two thresholds to THIRD-MID, and sites ≥ second threshold to THIRD-HIGH (Supplementary Data Tables S1 and S2). *Note:* 266 samples in the ENCODE dataset and 2 samples in the ATLAS dataset could not be divided into three groups by methylation value.

### 4.3. Data Analysis

For each sample and for each group of CpG-containing tetranucleotides, we first computed the total count of each tetranucleotide in the group. Next, we calculated the percentage difference between counts of reverse complementary tetranucleotides.

Formally, let X and Y be a pair of reverse complementary tetranucleotides, where X is the reverse complement of Y and vice versa, with X lexicographically less than Y. The percentage difference is defined as DIFF% = 200 × (*#X* − *#Y*)/(*#X + #Y*), where *#X* and *#Y* are counts of tetra-nucleotides X and Y in a given group, respectively.

Thus, DIFF% expresses the difference between the two counts as a percentage of their average. We used DIFF% as a metric of deviation from zero for reverse complementary patterns: DIFF% = 0 indicates perfect compliance with Chargaff’s second parity rule (CSPR).

We generated histograms with normal distribution fit curves to visualize the distribution of DIFF% values across all samples for each of the six pairs of reverse complementary tetranucleotides.

In addition, we calculated combinatorial probabilities of the observed distributions of counts for each pair of reverse complementary patterns. The counts of a tetranucleotide **X** and its reverse complement **X′** were modeled using the hypergeometric distribution. If the total counts of the two patterns are Xmax/X′max patterns of type X/X′ in each sample, respectively, and among them X and X′ patterns are randomly chosen, then the combinatorial probability is given by

$$P=\left(\begin{array}{c} Xmax\\ X \end{array}\right)*(\begin{array}{c} X\prime max\\ X\prime \end{array})/(\begin{array}{c} Xmax+X\prime max\\ X+X\prime \end{array}).$$

where $\left(\begin{array}{c} n\\ k \end{array}\right)$ denotes the binomial coefficient (“n choose k”). By Vandermonde’s identity, the sum of probabilities over all possible count combinations equals 1.

To speed up computations, we computed the base-10 logarithm of probabilities using double precision arithmetic. Because the logarithm of a product or quotient reduces to a sum or difference of logarithms, the calculation simplifies to summing precomputed factorials. This approach avoids the need for arbitrary-precision arithmetic methods.

Finally, using histogram-based normal distribution fits, we identified **outlier samples** within each group (excluding ALL), defined as samples with values exceeding ±3σ from the group mean. For each outlier sample and for each pair of reverse complementary tetra-nucleotides, we performed random sampling simulations to test whether similar or larger DIFF% values could arise by chance.

Specifically, for each outlier, we randomly selected (without replacement) the same number of CpG sites as in the sample (equal to the “TOTAL” column) from the entire CpG dataset. DIFF% values were computed for all six pairs in the simulated subset and compared to the outlier sample values. The simulation was terminated once all pairs produced a larger DIFF% than in the sample, or after at least 50,000 iterations.

All plots were created using MathWorks MATLAB R2023B—academic licenseAB.

## 5. Conclusions

Computational analysis of the two datasets revealed significant deviations of DNA methylation patterns from CSPR. The astronomically small probabilities, together with computer-based random sampling simulations of the observed statistical parameters, are analogous to **Levinthal’s paradox** in protein folding: random de novo DNA methylation could not realistically give rise to the observed methylation patterns within any biologically reasonable timescale, implying the involvement of non-random factors in the establishment of methylation marks.

Analysis of the distributions of deviations from CSPR in mutually reverse-complementary tetranucleotide pairs reveals a mirror asymmetry, formed by two non-overlapping mirror groups defined by CpG methylation values. This methylation asymmetry plays a compensatory role with respect to CSPR, as the deviations are reduced when the two groups are considered together. We identified four sequence motifs with two flanking purines that display pronounced asymmetry and two additional motifs that show reduced asymmetry.

We propose that methylation strand asymmetry emerges during cell differentiation, driven by the activity of epigenetic writers acting on the complex three-dimensional landscape of folded chromosomal DNA.

Furthermore, the proposed set of sample outliers may provide a useful resource for investigating strand-specific methylation patterns across distinct cell types.

## Figures and Tables

**Table 1:** Occurrence of reverse-complementary tetra-nucleotide pairs (with CpG in the middle) in (**A**) the human genome assembly GRCh37 (hg19), (**B**) the Human Methylome ATLAS dataset, and (**C**) the Human ENCODE dataset.

(**A**)															
		**chr:1–22**					**chr:X,Y**					**MT ^a^**			
**X**	**RC(X)**	**#X**		**#RC(X)**		**DIFF% ^b^**	**#X**	**#RC(X)**		**DIFF%**		**#X**	**#RC(X)**		**DIFF%**
ACGA	TCGT	1,499,090		1,507,388		0.55	90,947	91,596		0.71		40	19		71.19
ACGC	GCGT	1,540,984		1,547,980		0.45	78,385	78,482		0.12		38	10		116.67
ACGG	CCGT	1,720,518		1,722,715		0.13	90,846	90,739		0.12		20	28		33.33
CCGA	TCGG	1,639,565		1,643,109		0.22	87,349	87,388		0.04		37	26		34.92
CCGC	GCGG	1,961,235		1,958,592		0.13	90,322	91,009		0.76		53	11		131.25
GCGA	TCGC	1,396,774		1,396,553		0.02	74,398	74,104		0.40		16	47		98.41
ACGT		2,023,655					1 × 10^5^					21			
CCGG		2,191,202					1 × 10^5^					23			
GCGC		1,582,711					70,045					17			
TCGA		1,420,622					89,760					29			
(**B**)															
	**#X and #RC(X)**		**ACGA-TCGT (DIFF%)**		**ACGC-GCGT (DIFF%)**		**ACGG-CCGT (DIFF%)**		**CCGA-TCGG (DIFF%)**		**CCGC-GCGG (DIFF%)**			**GCGA-TCGC (DIFF%)**	
MAX	26,090,423		0.59		0.53		0.18		0.24		0.39			0.08	
MIN	24,869,777		0.54		0.40		0.13		0.20		0.08			0.06	
AVG	25,806,631		0.56		0.48		0.16		0.22		0.18			0.07	
STD	133,669		0.01		0.02		0.01		0.01		0.05			0.03	
(**C**)															
			**ACGA-TCGT**		**ACGC-GCGT**		**ACGG-CCGT**		**CCGA-TCGG**		**CCGC-GCGG**			**GCGA-TCGC**	
MAX	1,989,310		1.94		2.31		3.06		0.95		1.58			1.43	
MIN	938,201		1.26		0.90		1.16		0.39		0.86			0.07	
AVG	1,213,363		1.56		1.59		1.97		0.67		1.21			0.75	
STD	144,722		0.58		0.25		0.32		0.24		0.14			0.27	

^a^ MT denotes the mitochondrial genome; ^b^ DIFF% denotes the percentage difference between two numbers, calculated as their difference divided by their average, expressed as a percentage. We use DIFF% as a metric to estimate the divergence from zero for pairs of reverse complementary tetranucleotides. A DIFF% value of zero indicates perfect conformity to Chargaff’s second parity rule (CSPR).

**Table 2:** Deviations from Chargaff’s second parity rule (CSPR) between methylated reverse complementary tetranucleotide pairs (with 5mC in CpG context) in the Human Methylome Atlas dataset.

Statistical Functions	#X and #RC(X)	AC^m^GA-TC^m^GT (DIFF%)	AC^m^GC-GC^m^GT (DIFF%)	AC^m^GG-CC^m^GT (DIFF%)	CC^m^GA-TC^m^GG (DIFF%)	CC^m^GC-GC^m^GG (DIFF%)	GC^m^GA-TC^m^GC (DIFF%)
MAX	13,016,249	6.16	1.58	4.00	2.41	1.52	3.88
MIN	12,177,657	1.54	0.03	0.22	0.18	0.04	0.14
AVG	12,825,734	3.49	0.57	2.00	1.06	0.76	1.42
STD	108,100	0.94	0.24	0.76	0.38	0.27	0.71
**Cell type**							
1. Bone marrow Erythrocyte progenitors	13,016,249	2.73	1.19	1.53	0.18	0.81	0.59
2. Saphenous Vein Endothel	12,861,254	3.99	0.76	2.61	1.05	0.84	1.46
3. Blood T CD3	12,895,194	3.49	0.89	2.13	0.82	0.71	1.27
4. Pancreas Delta	12,900,680	3.19	0.45	2.00	1.11	0.72	1.44
5. Lung Pleura	12,856,313	3.45	0.33	1.84	1.16	0.77	1.33
6. Lung Alveolar Epithelial	12,833,368	3.95	0.65	2.53	1.06	0.80	1.26
7. Oligodendrocytes	12,648,071	1.54	1.10	0.22	1.15	0.32	0.14
8. Pancreas Duct	12,907,994	3.56	0.03	2.17	1.57	0.90	1.74
9. Neuron	12,682,753	1.98	1.58	0.36	1.78	1.09	0.27
10. Cortex Neuron	12,813,904	5.62	0.65	3.47	2.41	1.07	3.17
11. Cortex Neuron	12,875,201	3.04	1.15	1.54	1.00	0.04	0.95
12. Cortex Neuron	12,854,309	5.17	0.57	3.33	1.77	0.99	2.86
13. Prostate Epithelial	12,853,377	3.72	0.72	2.38	0.88	0.84	1.42
14. Blood T CenMem CD4	12,861,320	3.56	0.57	2.12	1.03	0.69	1.44
15. Blood T Naive CD8	12,776,669	6.16	0.54	4.00	2.02	1.52	3.88
16. Liver Endothelium	12,965,155	2.75	0.87	1.36	0.62	0.50	0.57

**Table 3:** Deviations from Chargaff’s second parity rule (CSPR) between non-methylated reverse complementary tetra-nucleotide pairs (with regular cytosine in CpG context) in the Human Methylome Atlas dataset.

Statistical Functions	#X and #RC(X)	ACGA-TCGT (DIFF%)	ACGC-GCGT (DIFF%)	ACGG-CCGT (DIFF%)	CCGA-TCGG (DIFF%)	CCGC-GCGG (DIFF%)	GCGA-TCGC (DIFF%)
MAX	13,157,044	3.45	1.12	3.50	1.88	1.69	4.79
MIN	12,692,120	0.09	0.01	0.14	0.00	0.04	0.17
AVG	12,984,243	1.19	0.41	1.36	0.58	0.65	2.05
STD	76,609	0.73	0.25	0.64	0.36	0.36	0.96
**Cell type**							
1. Bone marrow Erythrocyte progenitors	13,074,174	0.57	0.23	1.08	0.24	0.24	0.76
2. Saphenous Vein Endothel	13,072,709	1.41	0.11	1.80	0.56	0.70	2.10
3. Blood T CD3	12,939,424	0.83	0.09	1.72	0.35	0.88	1.81
4. Pancreas Delta	12,951,220	1.00	0.54	1.30	0.54	0.51	2.22
5. Lung Pleura	12,936,829	1.10	0.69	1.20	0.65	0.75	2.08
6. Lung Alveolar Epithelial	13,126,680	1.41	0.21	1.62	0.56	0.63	2.04
7. Oligodendrocytes	13,104,960	0.09	0.35	0.15	0.62	0.92	0.17
8. Pancreas Duct	12,914,646	1.35	1.12	1.46	0.99	0.88	2.68
9. Neuron	13,092,479	0.54	0.82	0.64	1.26	1.69	0.41
10. Cortex Neuron	12,960,157	3.45	0.30	2.78	1.88	0.75	3.97
11. Cortex Neuron	12,890,916	1.06	0.41	0.97	0.52	0.50	1.42
12. Cortex Neuron	12,936,115	3.07	0.29	2.64	1.27	0.71	3.64
13. Prostate Epithelial	12,928,988	1.36	0.12	1.52	0.41	0.81	2.25
14. Blood T CenMem CD4	12,988,114	0.84	0.35	1.67	0.57	0.71	1.98
15. Blood T Naive CD8	13,008,722	3.29	0.39	3.50	1.64	1.45	4.79
16. Liver Endothelium	13,055,049	0.52	0.01	0.87	0.17	0.17	0.80

**Table 4:** Outlier samples showing deviations greater than three standard deviations (3σ) from the average value.

Cell Type	Number of Samples	Outlier in Groups
Blood Monocytes	1	ALL;
Blood T Naive CD4	1	THIRD-LOW;
Blood T Naive CD8	1	HALF-LOW; HALF-HIG; THIRD-LOW;
Bone marrow Erythrocyte progenitors	2	ALL; VALZERO; VALONE; THIRD-LOW; THIRD-MID; THIRD-HIG;
Colon Left Endocrine	1	ALL;
Cortex Neuron	3	ALL; VALONE; HALF-LOW; HALF-HIG; THIRD-MID; THIRD-HIG;
Fallopian Epithelial	1	ALL;
Neuron	1	VALONE; HALF-LOW; HALF-HIG; THIRD-LOW; THIRD-MID; THIRD-HIG;
Oligodendrocytes	1	VALONE; HALF-LOW; HALF-HIG; THIRD-MID; THIRD-HIG;
Pancreas Acinar	1	VALZERO

## Data Availability

The original contributions presented in this study are included in the article/Supplementary Materials. Further inquiries can be directed to the corresponding authors.

## Supplementary Materials

The following supporting information can be downloaded at: https://www.mdpi.com/article/10.3390/ijms26199504/s1.

## Abbreviations

The following abbreviations are used in this manuscript:

5mC	5-methylcytosine
CSPR	Chargaff’s Second Parity Rule
DIFF%	Percentage difference
MAX	Maximal value
MIN	Minimal value
AVG	Average of all values
STD	Standard deviation
