# Supplementary material for: Study of the Patterns of DNA Methylation in Human Cells Through the Prism of Intra-Strand DNA Symmetry
Source: Int J Mol Sci. 2025 Sep 28;26(19):9504. doi: 10.3390/ijms26199504 (PMC12524719; doi:10.3390/ijms26199504)

ATLAS group ALL

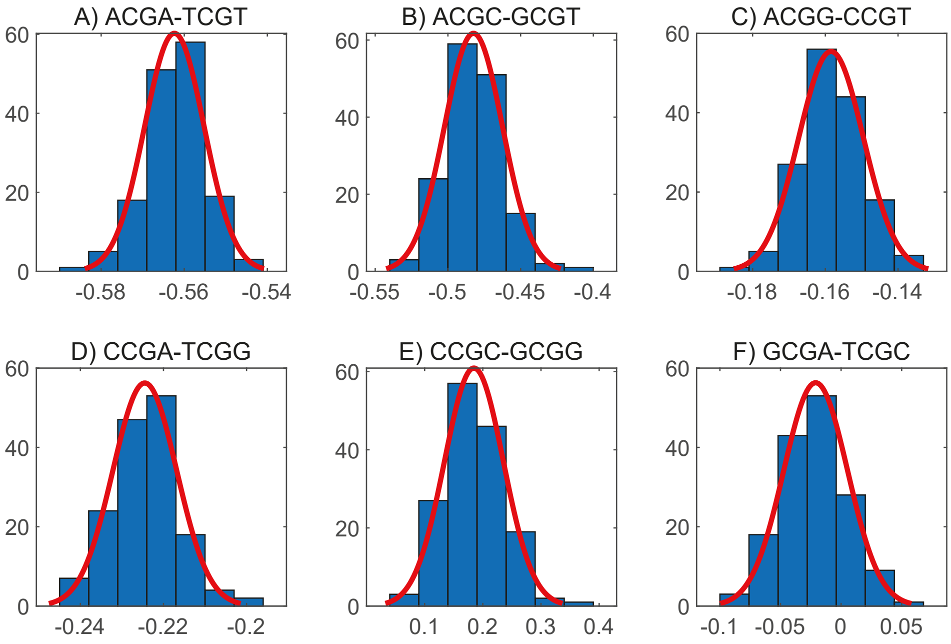

ATLAS group VALZERO

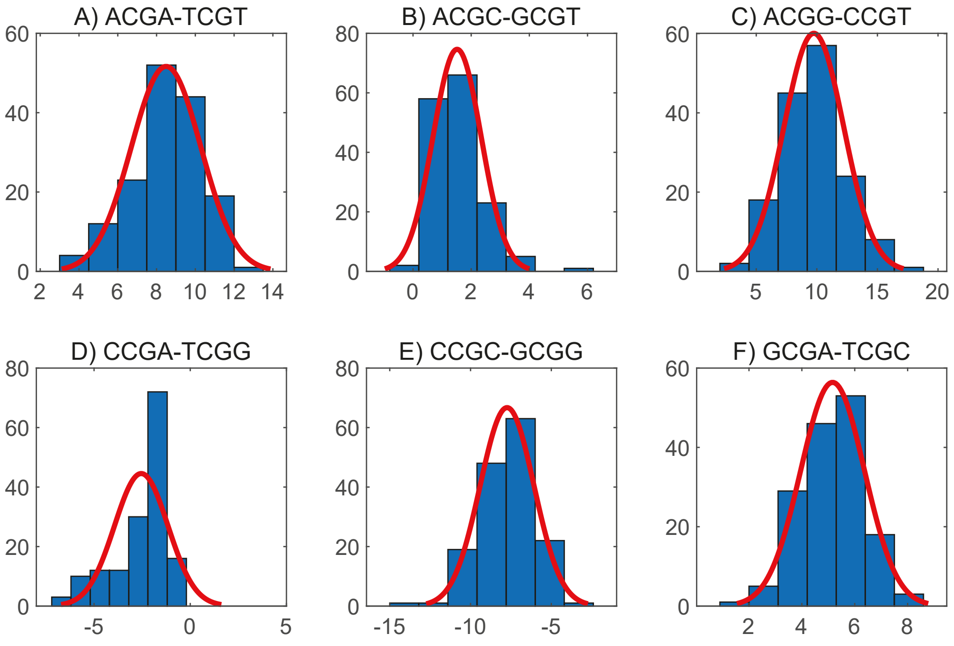

ATLAS group VALONE

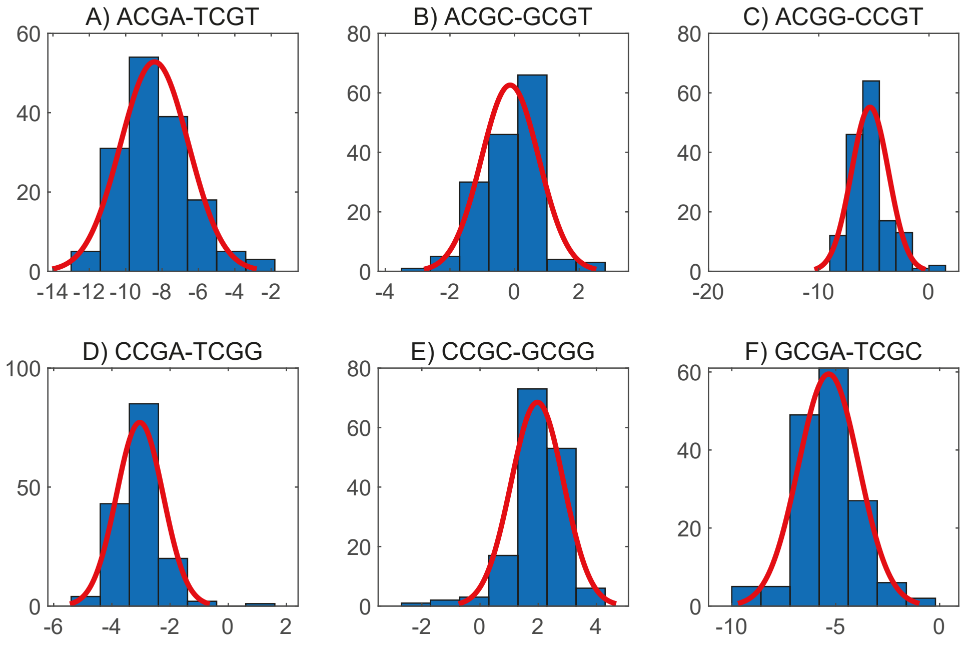

ATLAS group HALF-LOW

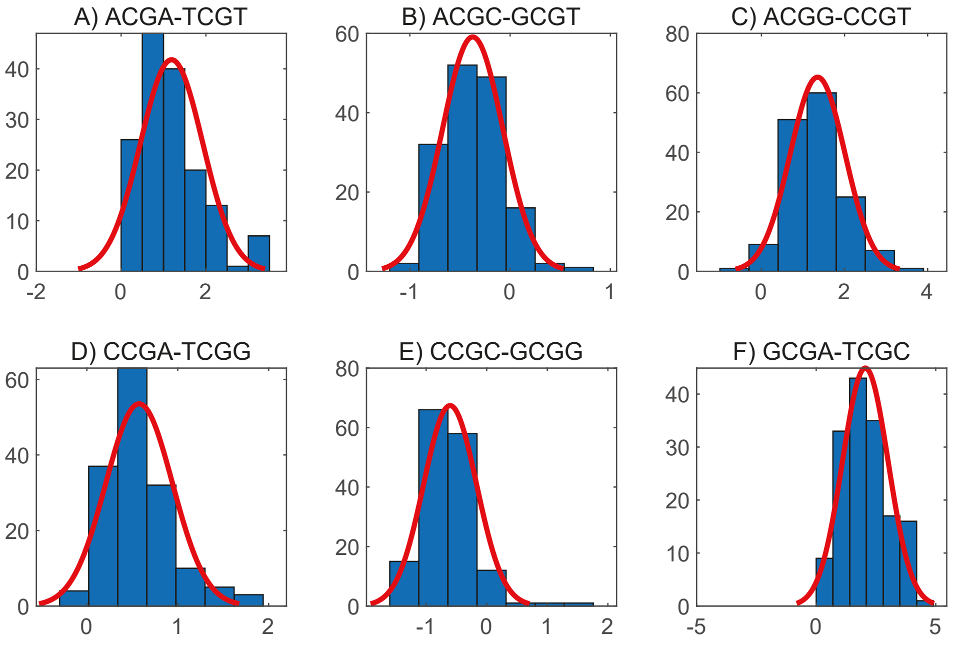

ATLAS group HALF-HIG

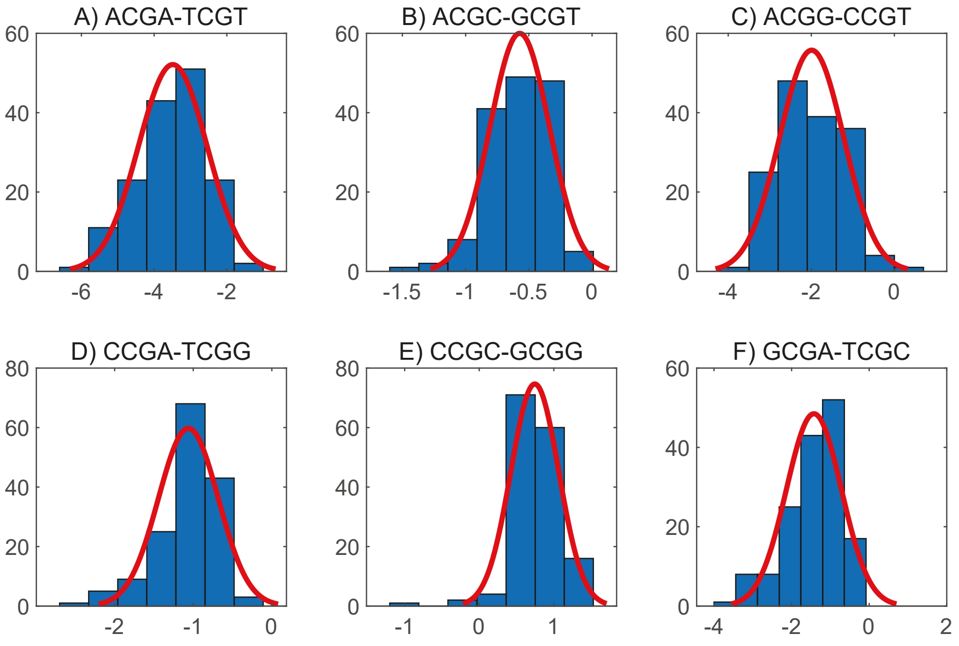

ATLAS group THIRD-LOW

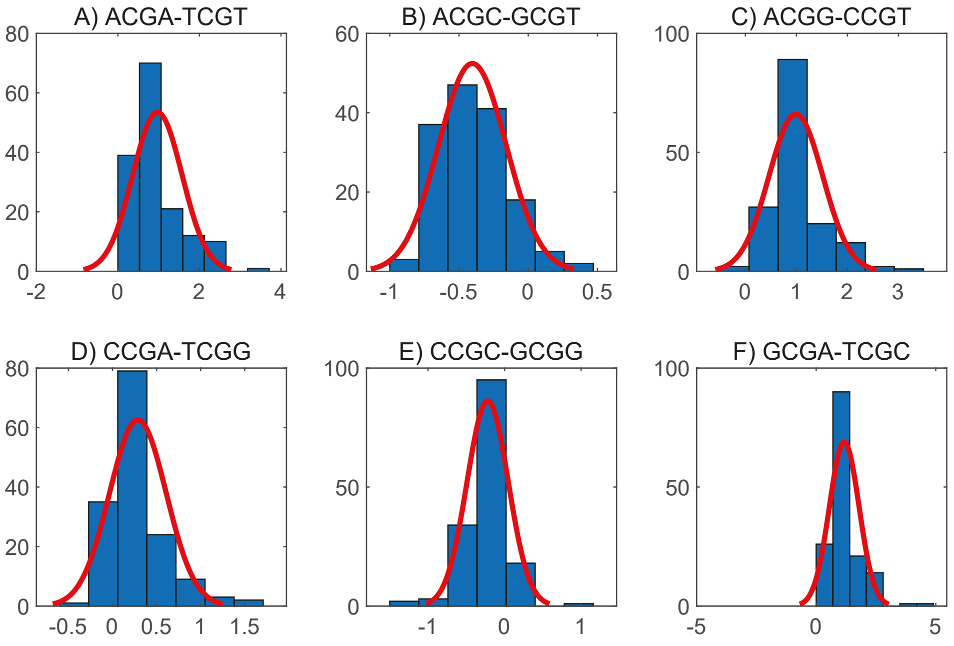

ATLAS group THIRD-MID

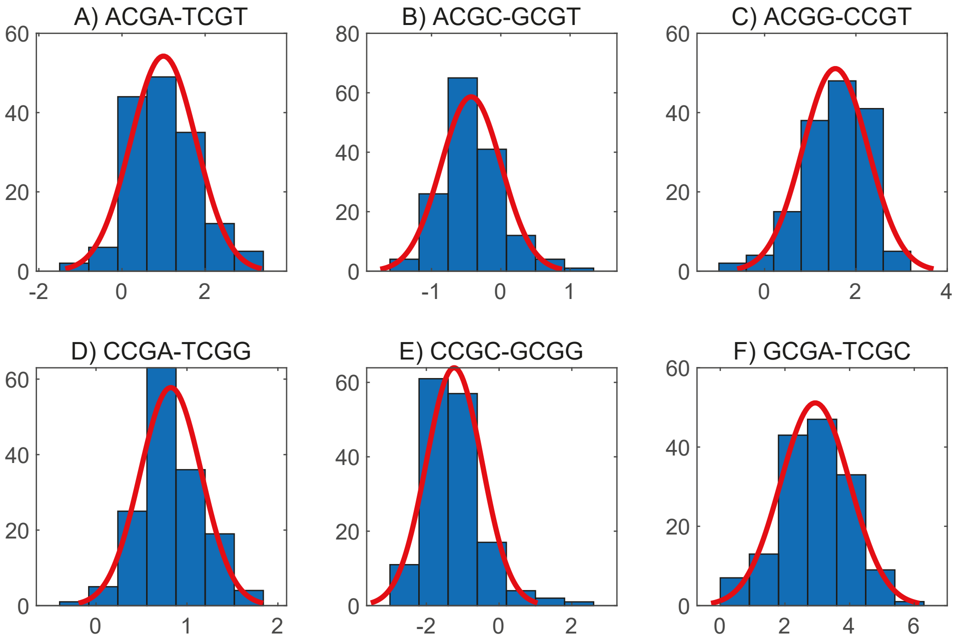

ATLAS group THIRD-HIG

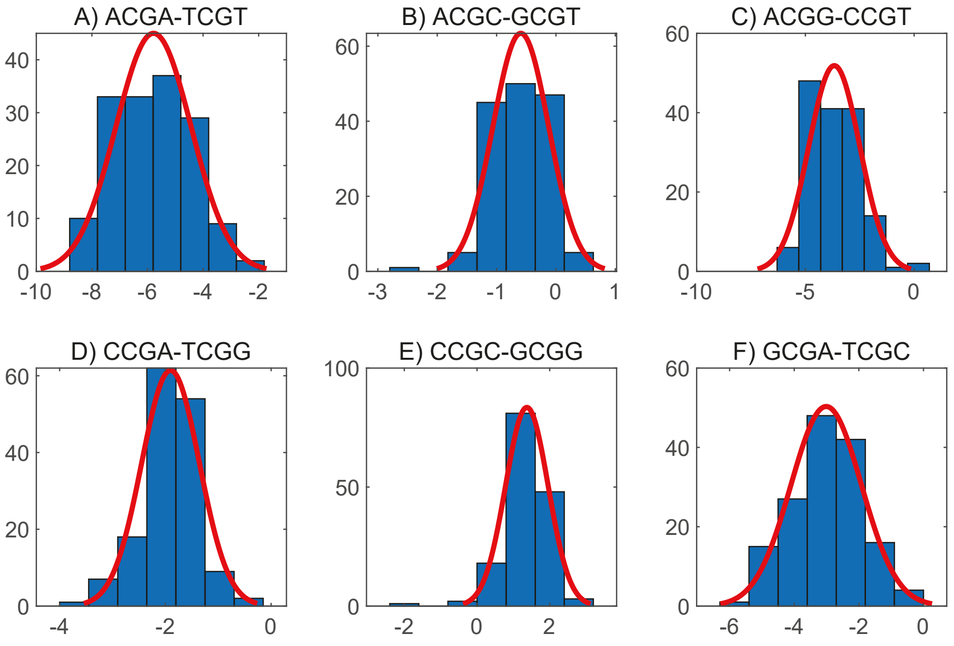

Supplement: Supplementary file 1 [file ijms-26-09504-s001.zip › Figure S1 - ATLAS.pdf]
