# Supplementary material for: Study of the Patterns of DNA Methylation in Human Cells Through the Prism of Intra-Strand DNA Symmetry
Source: Int J Mol Sci. 2025 Sep 28;26(19):9504. doi: 10.3390/ijms26199504 (PMC12524719; doi:10.3390/ijms26199504)

ENCODE group ALL

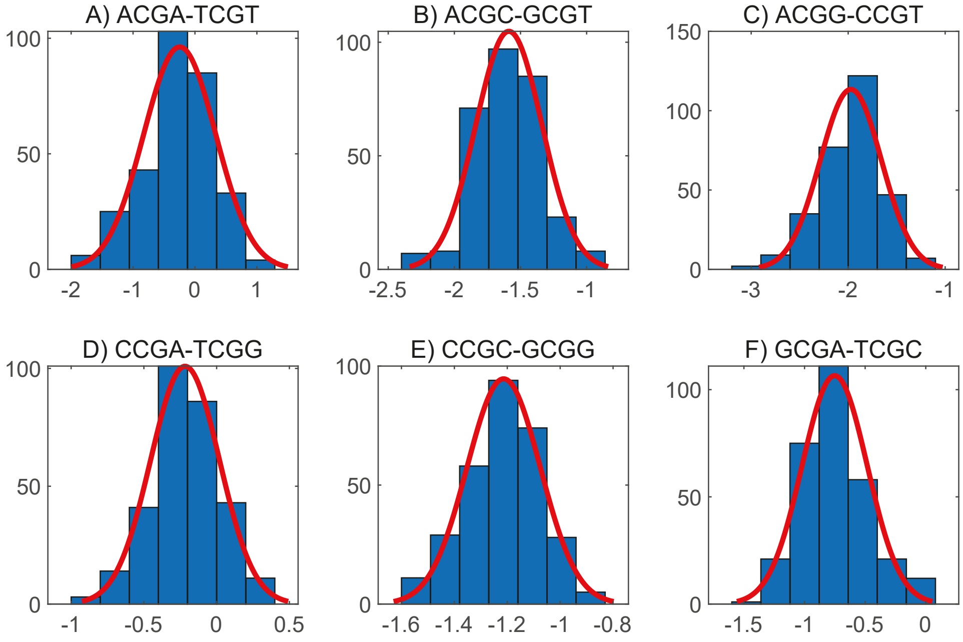

ENCODE group VALZERO

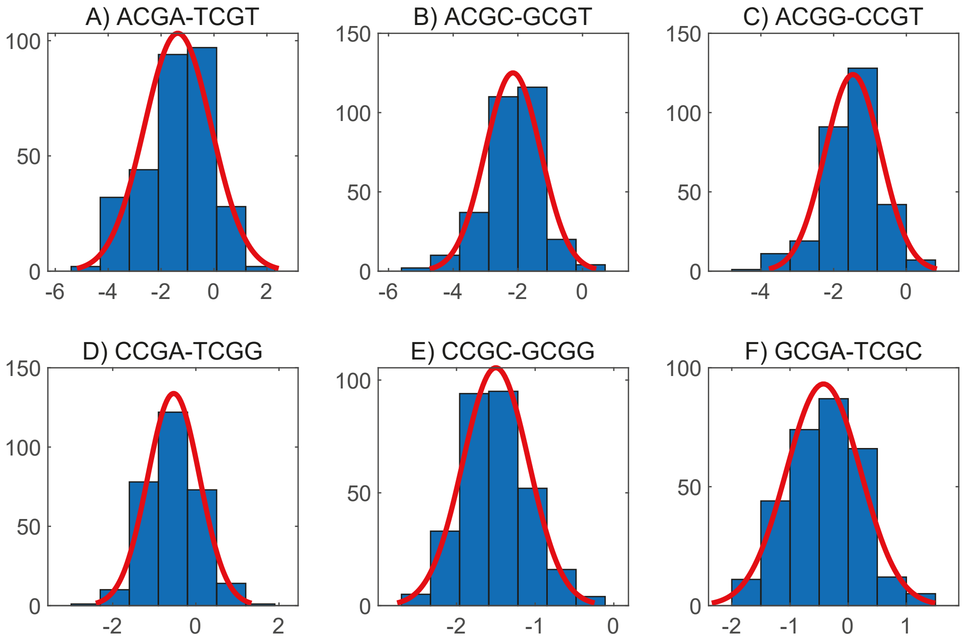

ENCODE group VALONE

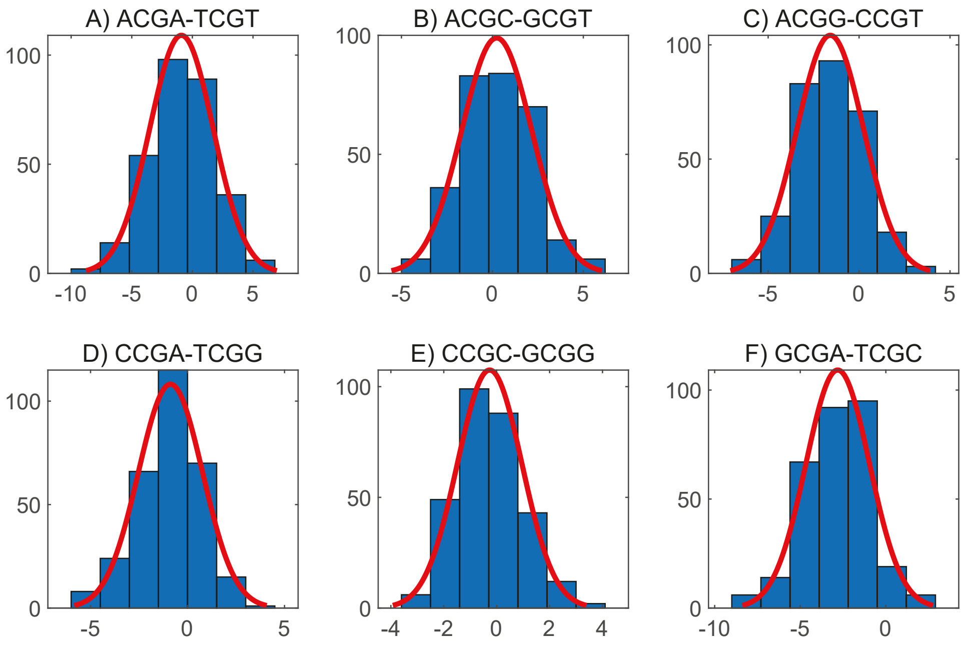

ENCODE group HALF-LOW

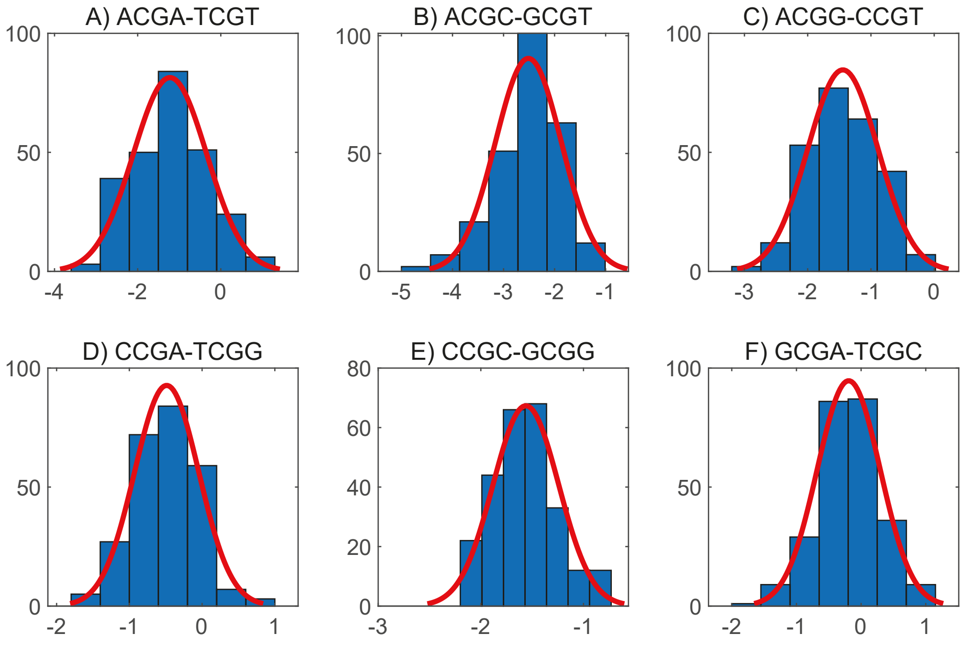

ENCODE group HALF-HIG

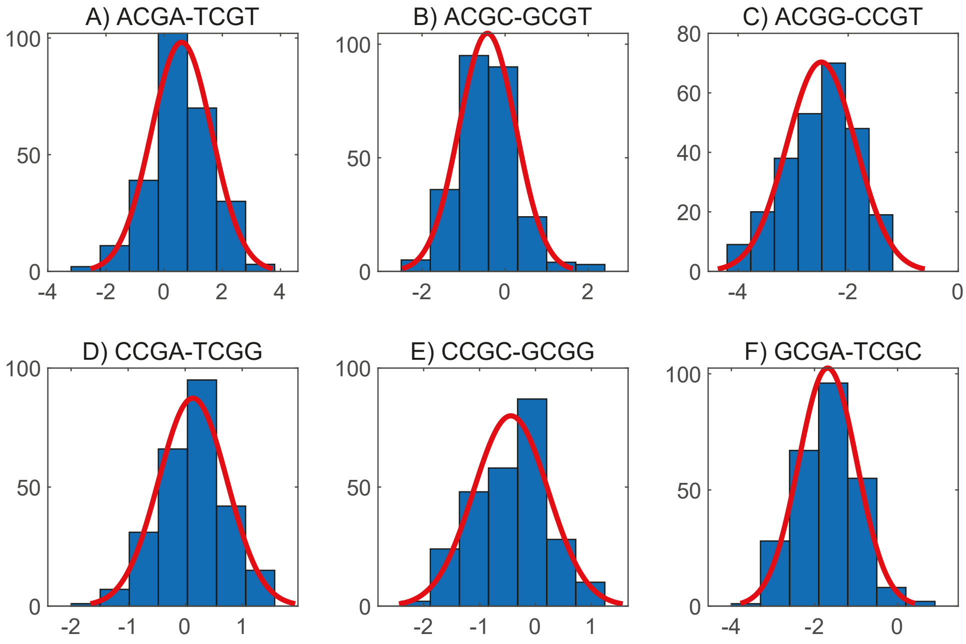

ENCODE group THIRD-LOW

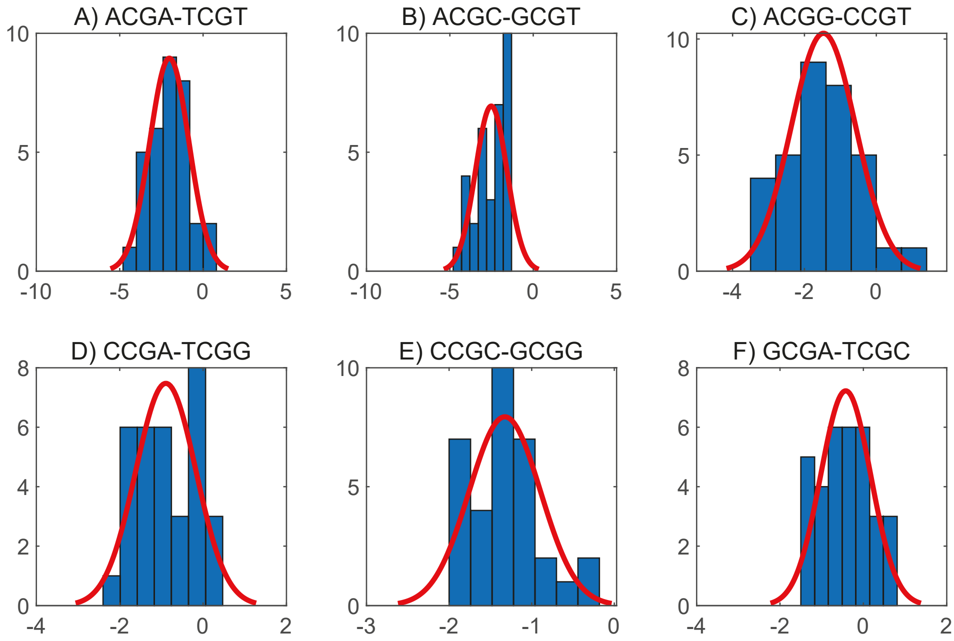

ENCODE group THIRD-MID

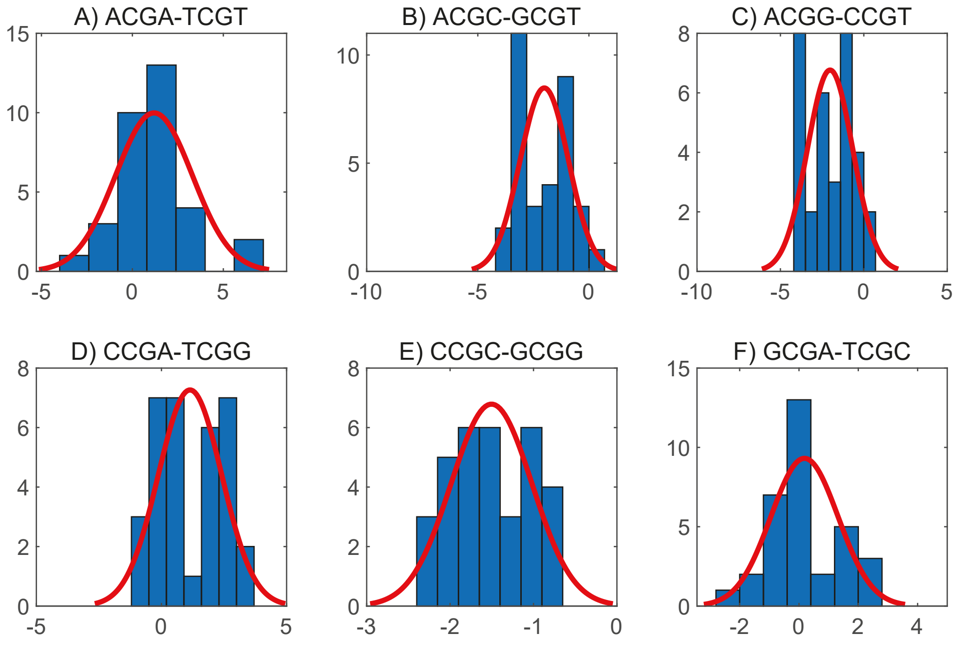

ENCODE group THIRD-HIG

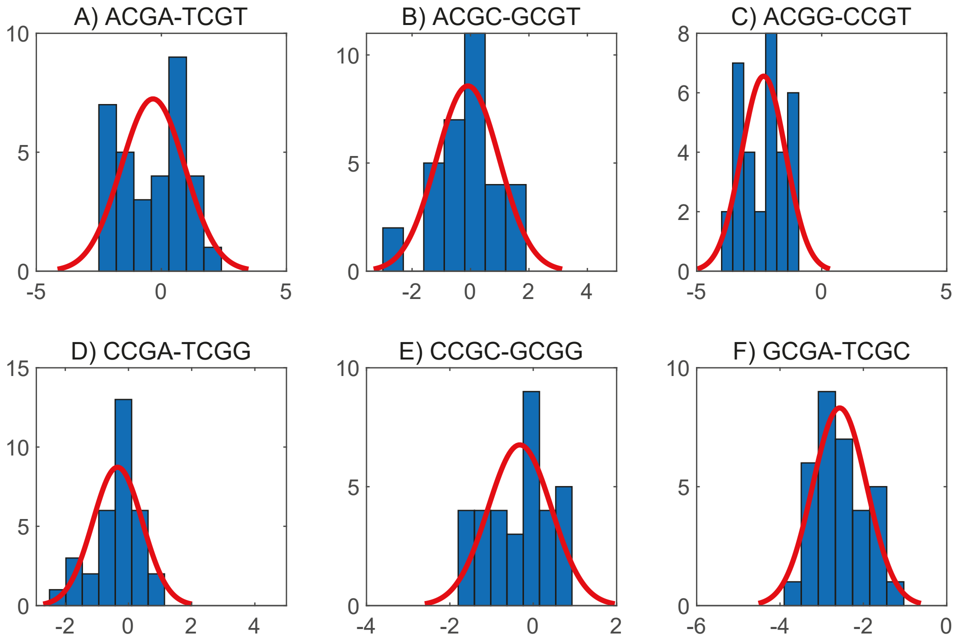

Supplement: Supplementary file 1 [file ijms-26-09504-s001.zip › Figure S2 - ENCODE.pdf]
