# Supplementary material for: Study of the Patterns of DNA Methylation in Human Cells Through the Prism of Intra-Strand DNA Symmetry
Source: Int J Mol Sci. 2025 Sep 28;26(19):9504. doi: 10.3390/ijms26199504 (PMC12524719; doi:10.3390/ijms26199504)
Supplement: Supplementary file 1 [file ijms-26-09504-s001.zip › Figure S3 - ATLAS diff vs probability.pdf]

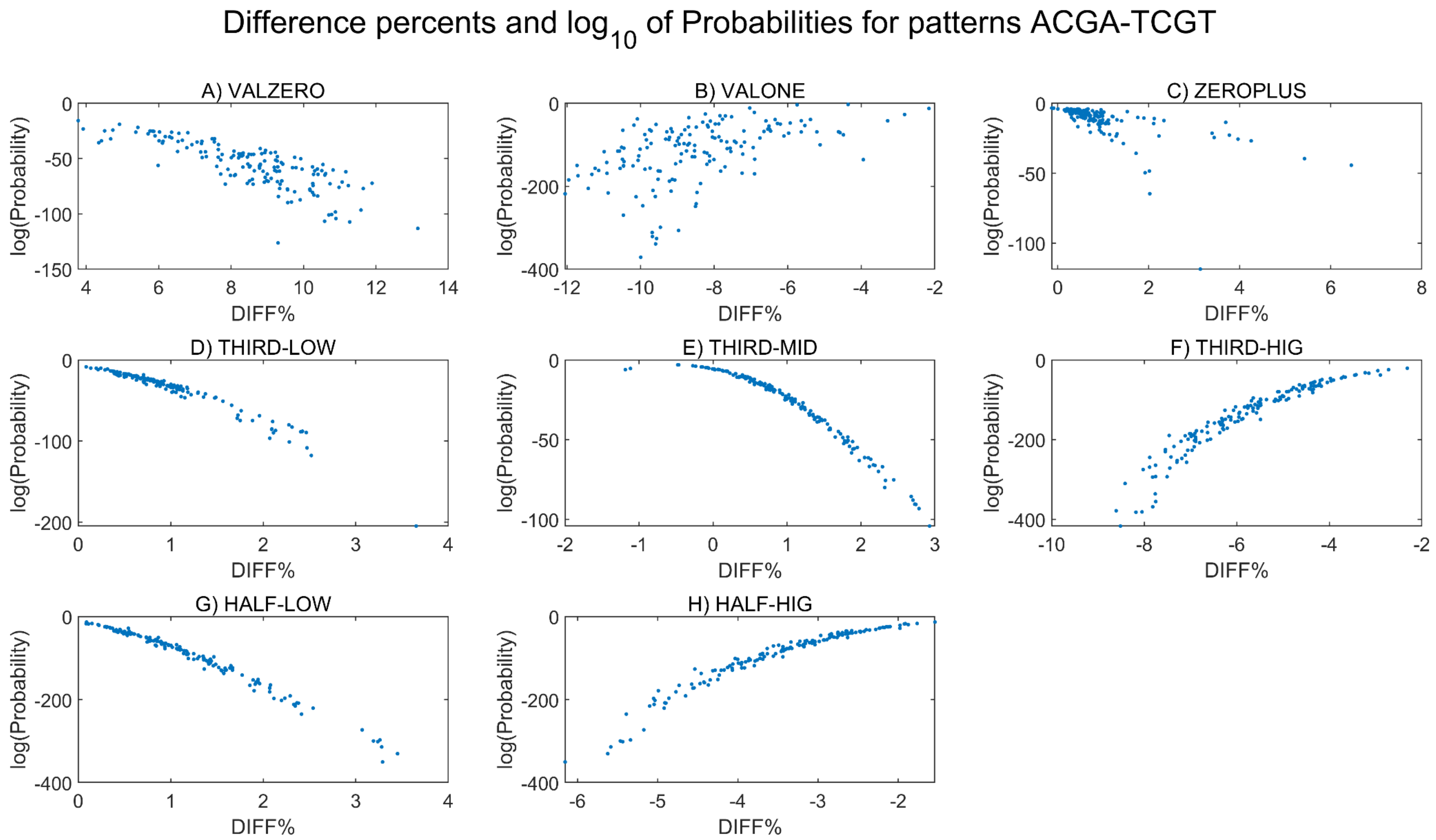
Difference percents and log10 of Probabilities for patterns ACGA-TCGT
A) VALZERO
B) VALONE
C) ZEROPLUS
D) THIRD-LOW
E) THIRD-MID
F) THIRD-HIG
G) HALF-LOW
H) HALF-HIG
log(Probability)
DIFF%
0
-50
-100
-150
-200
-400
4
6
8
10
12
14
-12
-10
-8
-6
-4
-2
0
1
2
3
-1
-2
-3
-4
-5
-6

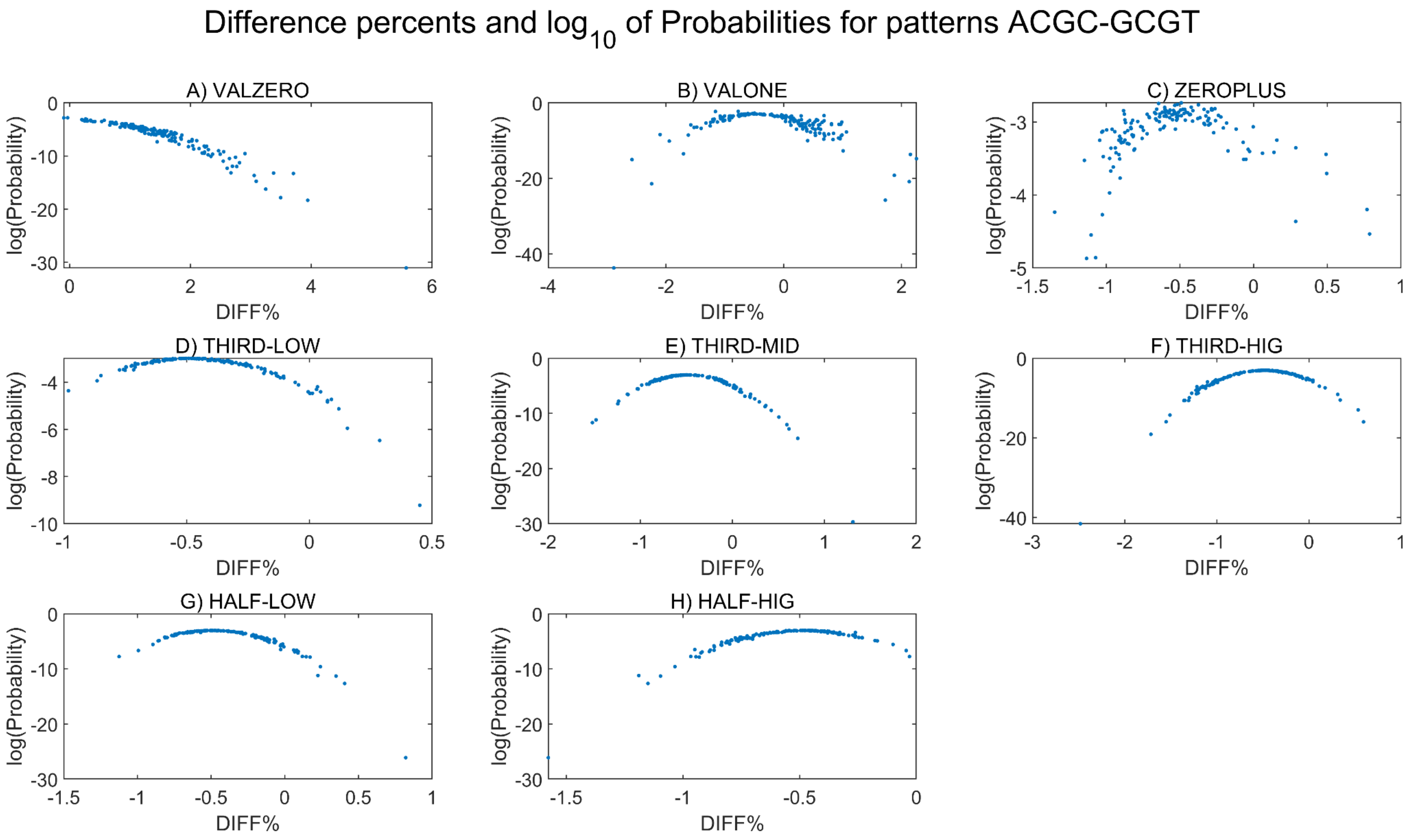
Difference percents and $\log_{10}$ of Probabilities for patterns ACGC-GCGT
A) VALZERO
B) VALONE
C) ZEROPLUS
D) THIRD-LOW
E) THIRD-MID
F) THIRD-HIG
G) HALF-LOW
H) HALF-HIG
log(Probability)
DIFF%

Difference percents and $\log_{10}$ of Probabilities for patterns ACGG-CCGT

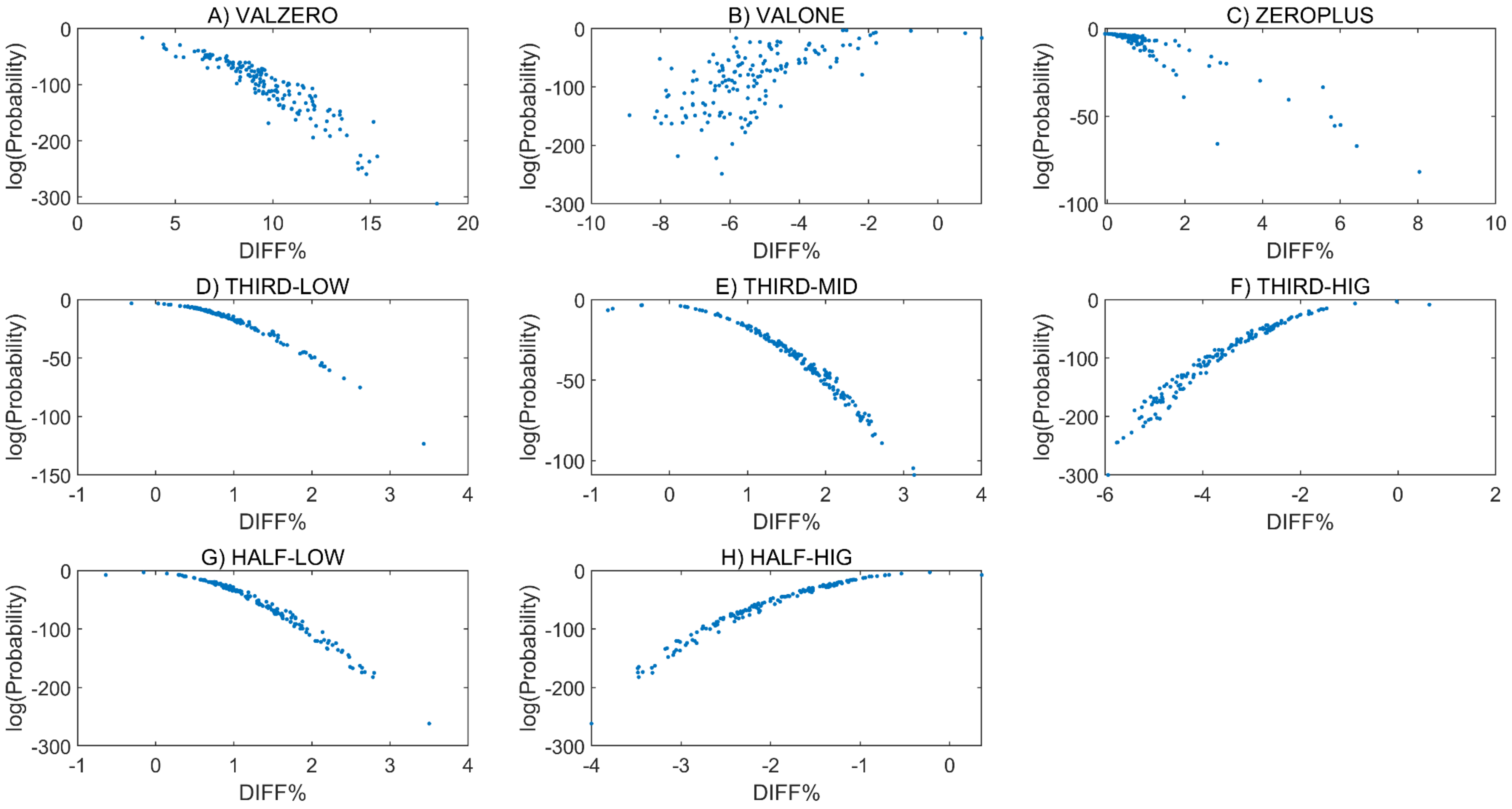

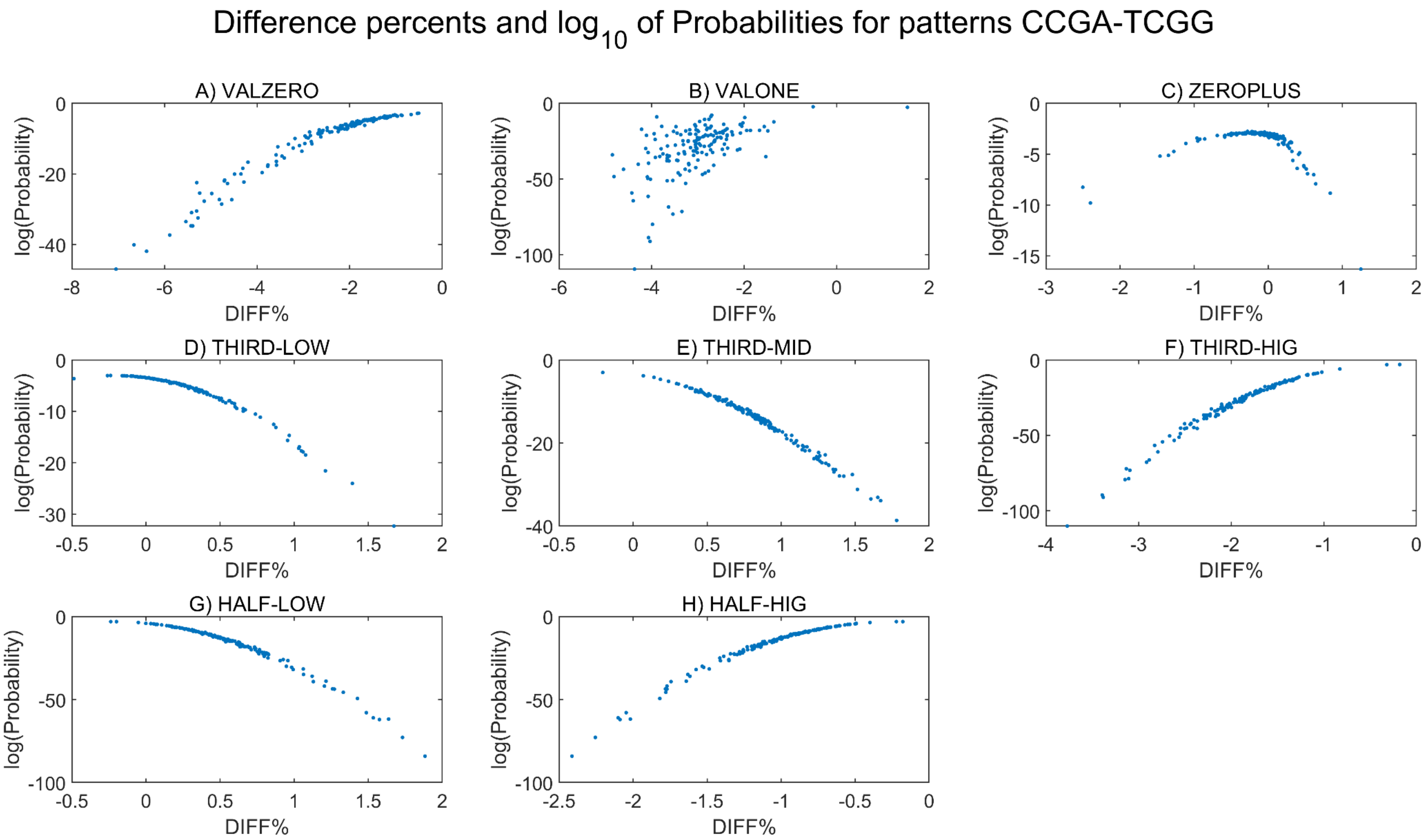
Difference percents and log10 of Probabilities for patterns CCGA-TCGG
A) VALZERO
B) VALONE
C) ZEROPLUS
D) THIRD-LOW
E) THIRD-MID
F) THIRD-HIG
G) HALF-LOW
H) HALF-HIG
log(Probability)
DIFF%

Difference percents and $\log_{10}$ of Probabilities for patterns CCGC-GCGG

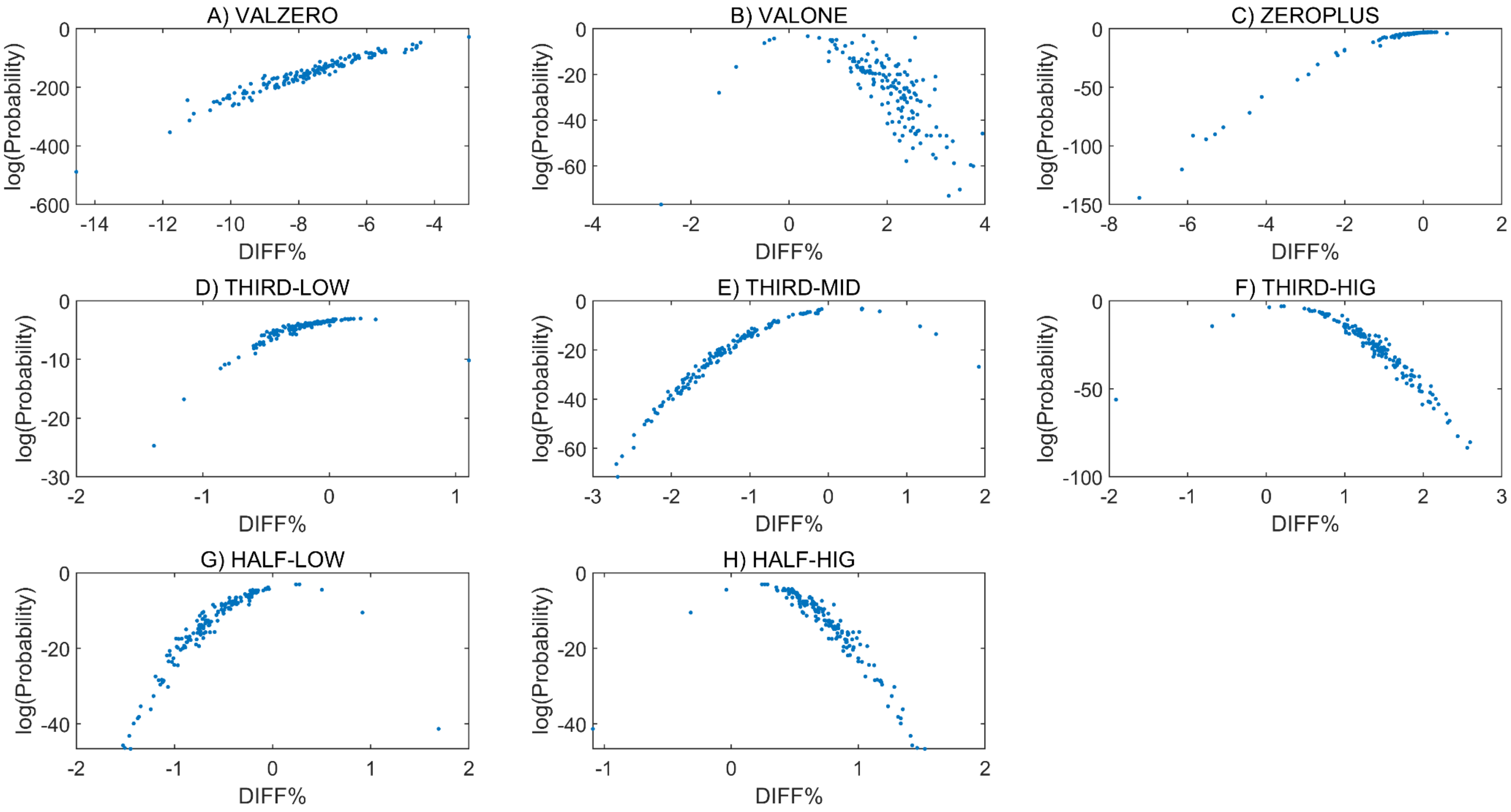

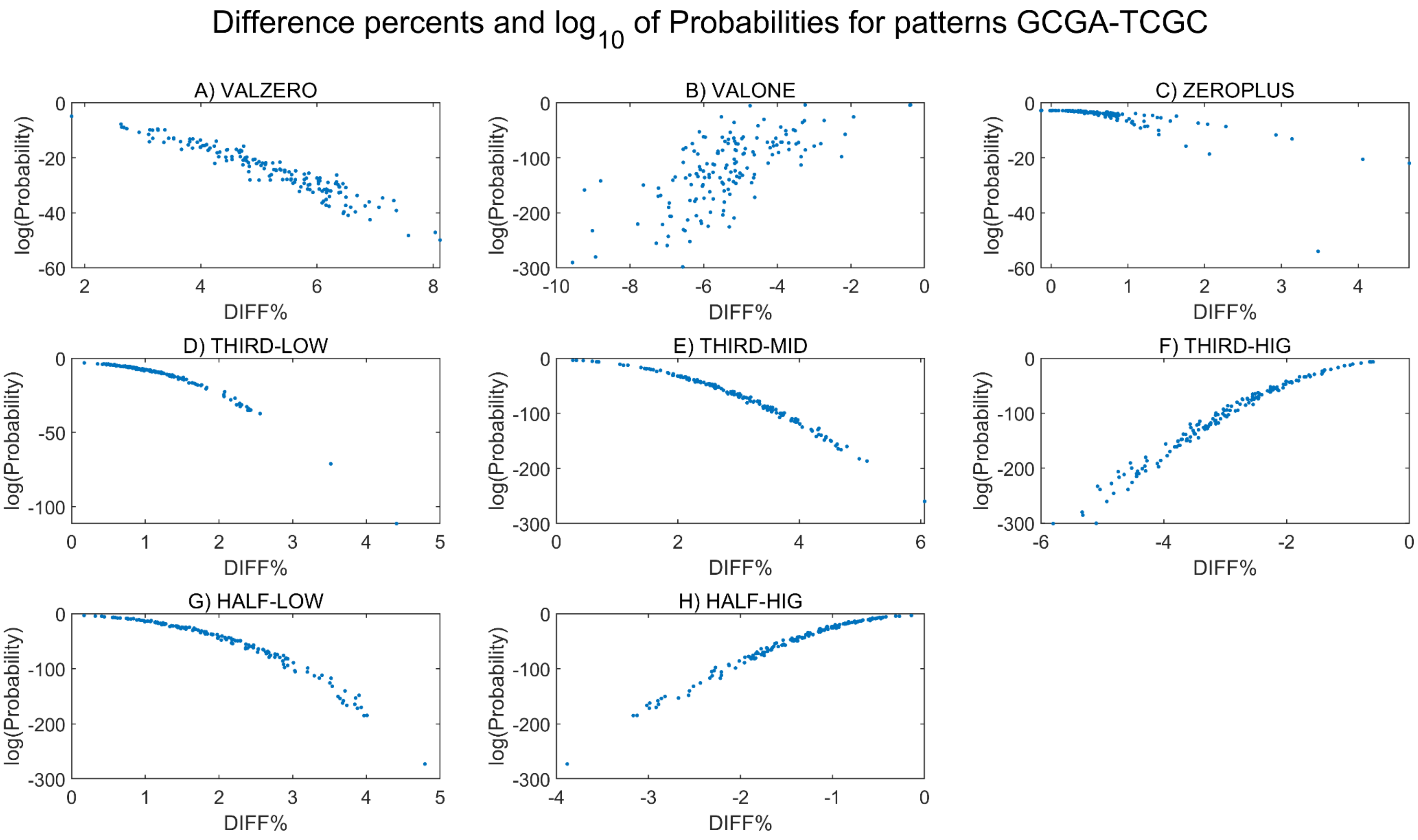

Difference percents and log10 of Probabilities for patterns GCGA-TCGC
A) VALZERO
B) VALONE
C) ZEROPLUS
D) THIRD-LOW
E) THIRD-MID
F) THIRD-HIG
G) HALF-LOW
H) HALF-HIG
log(Probability)
DIFF%
