# Supplementary material for: Study of the Patterns of DNA Methylation in Human Cells Through the Prism of Intra-Strand DNA Symmetry
Source: Int J Mol Sci. 2025 Sep 28;26(19):9504. doi: 10.3390/ijms26199504 (PMC12524719; doi:10.3390/ijms26199504)
Supplement: Supplementary file 1 [file ijms-26-09504-s001.zip › Figure S4 - ENCODE diff vs probability.pdf]

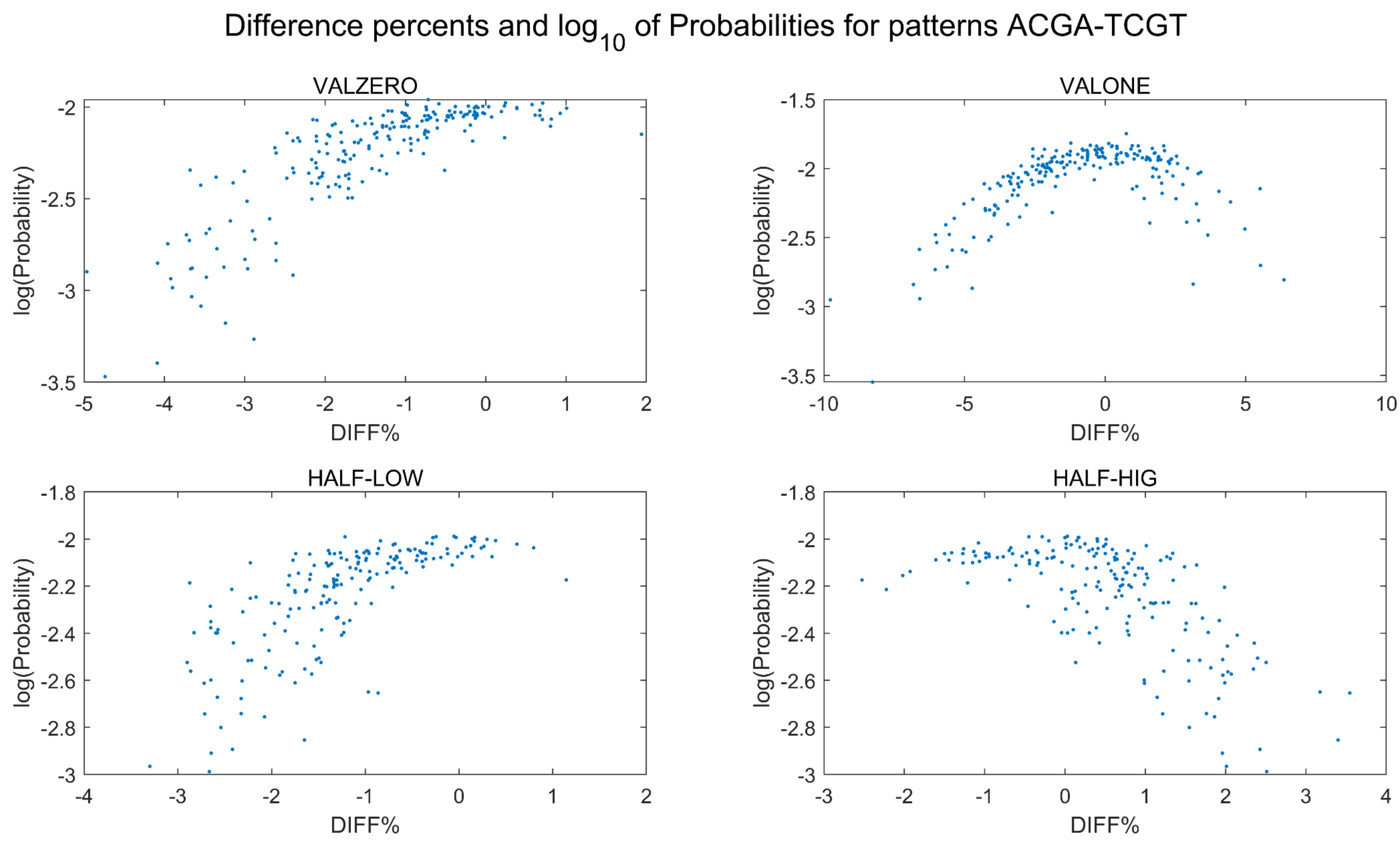
Difference percents and log_{10} of Probabilities for patterns ACGA-TCGT
VALZERO
VALONE
HALF-LOW
HALF-HIG
log(Probability)
DIFF%
-2
-2.5
-3
-3.5
-5
-4
-3
-2
-1
0
1
2
-1.5
-10
-5
0
5
10
-1.8
-2.2
-2.4
-2.6
-2.8
3
4

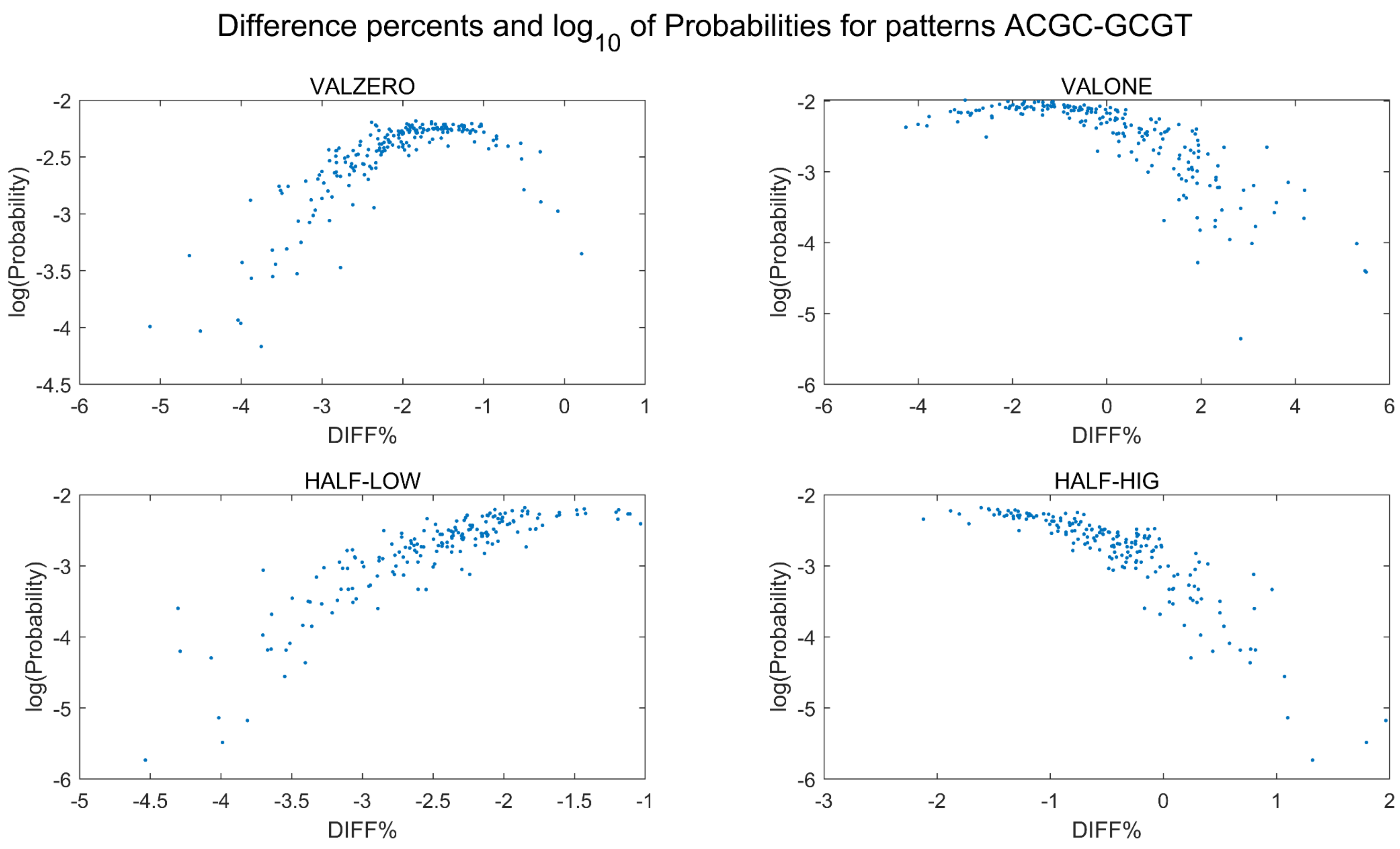
Difference percents and $\log_{10}$ of Probabilities for patterns ACGC-GCGT
VALZERO
VALONE
HALF-LOW
HALF-HIG
log(Probability)
DIFF%
-2
-2.5
-3
-3.5
-4
-4.5
-6
-5
-4
-3
-2
-1
0
1
2
4
6
-4.5
-3.5
-2.5
-1.5

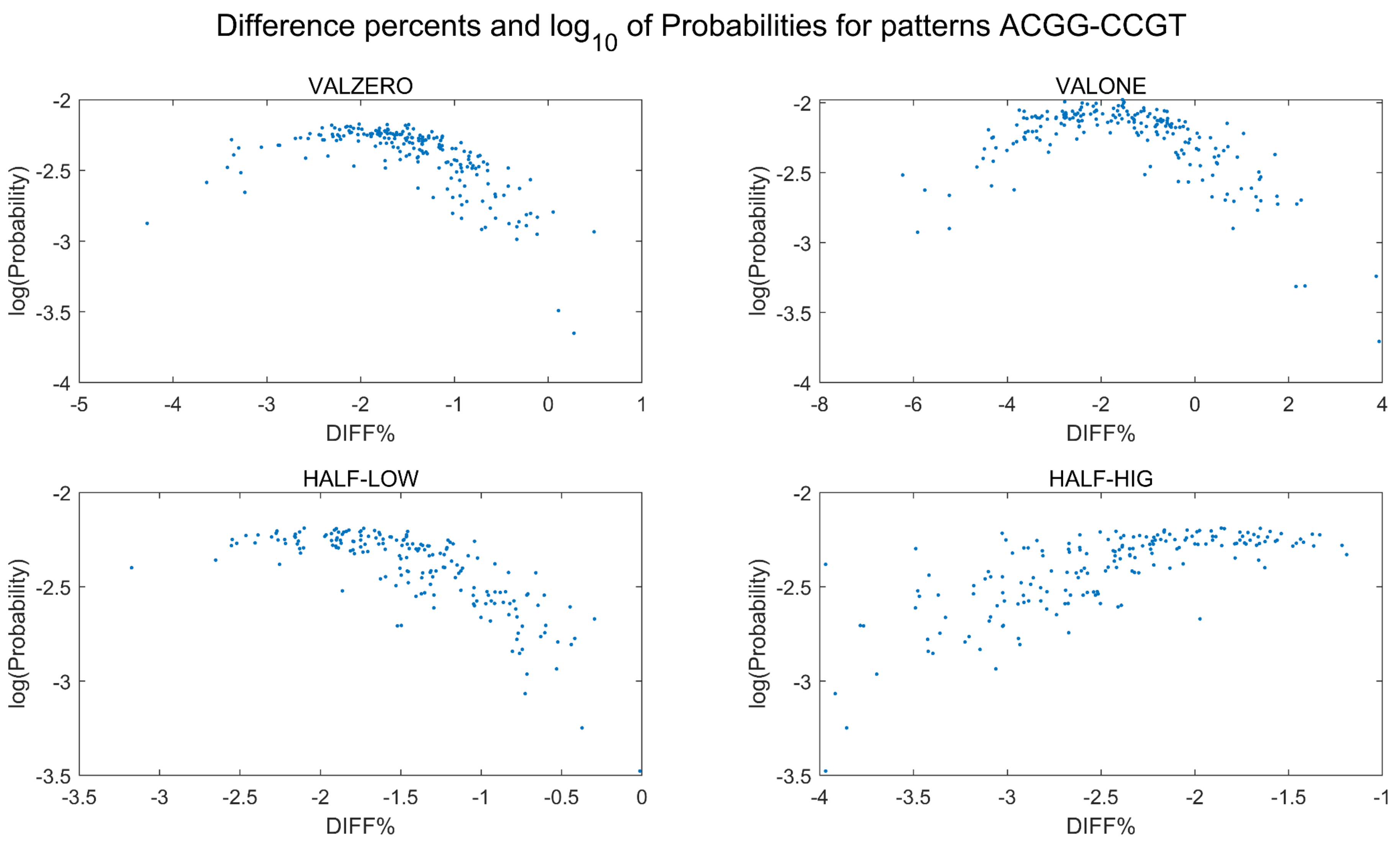
Difference percents and log10 of Probabilities for patterns ACGG-CCGT
VALZERO
VALONE
HALF-LOW
HALF-HIG
log(Probability)
DIFF%
-2
-2.5
-3
-3.5
-4
-5
-4
-3
-2
-1
0
1
-8
-6
-4
-2
0
2
4
-3.5
-3
-2.5
-2
-1.5
-1
-0.5
-4
-3.5
-3
-2.5
-2
-1.5
-1

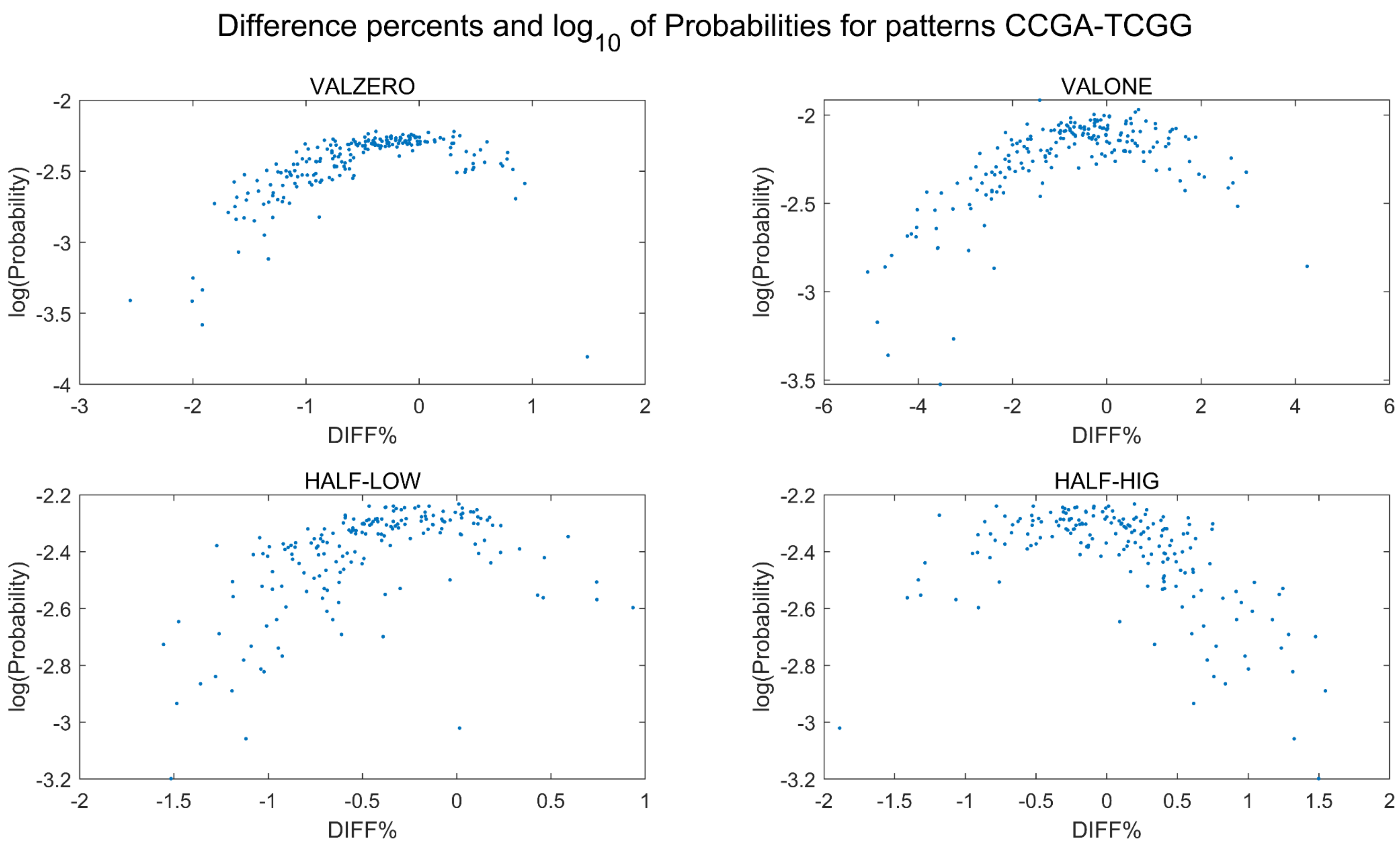
Difference percents and log_{10} of Probabilities for patterns CCGA-TCGG
VALZERO
VALONE
HALF-LOW
HALF-HIG
log(Probability)
DIFF%

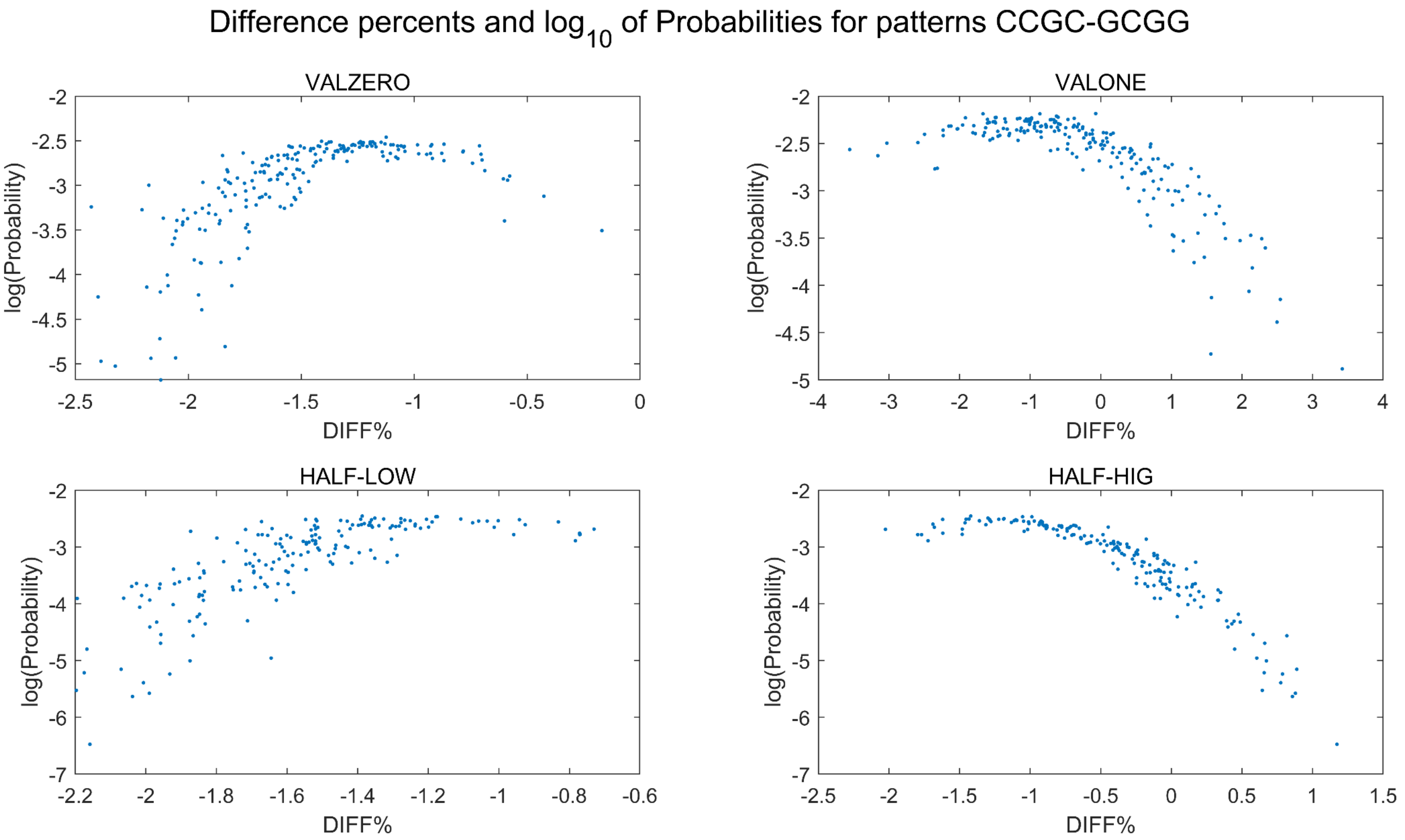

Difference percents and log10 of Probabilities for patterns CCGC-GCGG
VALZERO
VALONE
HALF-LOW
HALF-HIG
log(Probability)
DIFF%

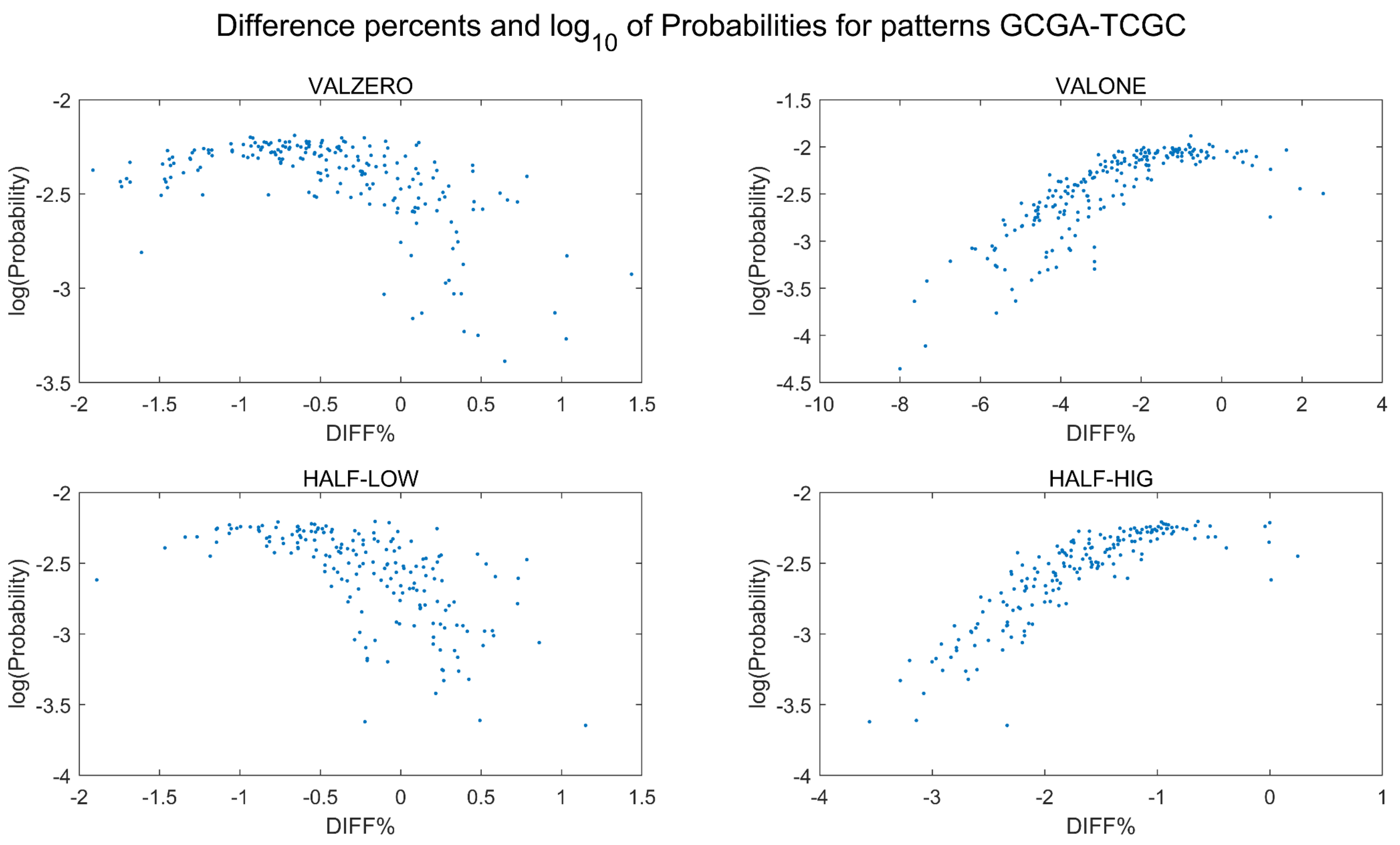
Difference percents and $\log_{10}$ of Probabilities for patterns GCGA-TCGC
VALZERO
VALONE
HALF-LOW
HALF-HIG
log(Probability)
DIFF%
